# Synthesis of axially chiral oxazoline–carbene ligands with an *N*-naphthyl framework and a study of their coordination with AuCl·SMe_2_

**DOI:** 10.3762/bjoc.8.81

**Published:** 2012-05-11

**Authors:** Feijun Wang, Shengke Li, Mingliang Qu, Mei-Xin Zhao, Lian-Jun Liu, Min Shi

**Affiliations:** 1 Key Laboratory for Advanced Materials and Institute of Fine Chemicals, East China University of Science and Technology, 130 MeiLong Road, Shanghai 200237, P. R. China; 2 State Key Laboratory of Organometallic Chemistry, Shanghai Institute of Organic Chemistry, Chinese Academy of Sciences, 354 Fenglin Road, Shanghai 200032, P. R. China

**Keywords:** axially chiral ligand, gold, *N*-heterocyclic carbenes, *N*-naphthyl framework

## Abstract

Axially chiral oxazoline–carbene ligands with an *N*-naphthyl framework were successfully prepared, and their coordination behavior with AuCl·SMe_2_ was also investigated, affording the corresponding Au(I) complexes in moderate to high yields.

## Introduction

**Figure 1:**
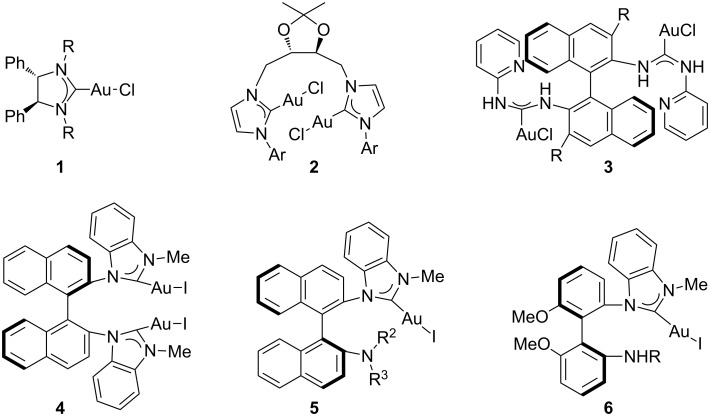
Chiral NHC–Au(I) complexes.

During the past decade, with an explosive growth of asymmetric homogeneous gold catalysis in C–C, C–O, or C–N bond formations, the design and synthesis of chiral gold complexes has received wide attention. Compared with the more commonly used air-sensitive phosphine ligand, *N*-heterocyclic carbenes (NHCs), with intrinsic characteristics such as strong δ-donor but poor π-acceptor abilities, ease of preparation, air and thermal stability of their metal complexes, and the convenient introduction of chiral elements, have also emerged as effective ligands for a number of homogeneous gold catalyzes [1–8]. However, during our ongoing survey of chiral NHC–Au(I) complexes in the literature, we only found a few unique papers of relevance. Tomioka and co-workers disclosed the first chiral NHC–Au(I) complex **1** (Figure 1), which was applied to catalyze the asymmetric cyclization of 1,6-enynes giving the corresponding cyclopentane derivatives with moderate enantioselectivity up to 59% [9–10]. Iglesias and co-workers reported a type of NHC–Au(I) complexes **2** containing *C*_2_-symmetric bis(NHC)-ligands with two imidazolin-2-ylidene moieties on a chiral dioxolane backbone, produced in up to 95% ee by hydrogenation of a prochiral alkene [11]. Recently, Toste and co-workers reported a novel family of axially chiral (acyclic diaminocarbene) gold(I) complexes **3** derived from 3,3′-substituted 1,1′-binaphthalenyl-2,2′-diamine, and their application in the dynamic kinetic asymmetric transformation of propargyl esters, giving the corresponding substituted chromenes in up to 99% ee [12]. Our group also developed a new family of axially chiral NHC–Au(I) complexes (**4**–**6**) with a binaphthyl or biphenyl framework [13–14]. These Au(I) complexes were applied to catalyze the asymmetric cyclization of 1,6-enynes or allene in up to 70% ee, and the asymmetric intramolecular hydroamination of allene in up to 44% ee.

**Figure 2:**
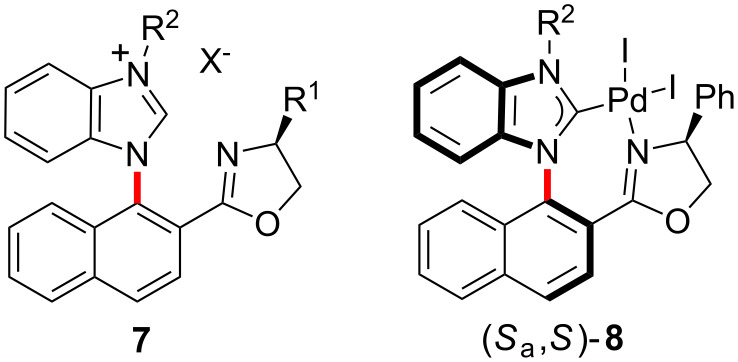
Axially chiral oxazoline–carbene ligands and their palladium complexes.

We previously reported a novel type of axially chiral ligand **7** with an *N*-naphthyl framework (Figure 2) instead of traditional binaphthyl framework [15]. Their palladium complexes **8** showed high stereoselectivities in asymmetric allylic arylations to achieve the kinetic resolution of Morita–Baylis–Hillman adducts, affording up to 99% ee of the (*E*)-allylation products and 92% ee of the recovered Morita–Baylis–Hillman adducts. These intriguing results stimulated us to further develop the axially chiral oxazoline–carbene ligands **7** with an *N*-naphthyl framework and to evaluate their coordination with AuCl·SMe_2_.

## Results and Discussion

### Synthesis of axially chiral ligands

**Scheme 1:**
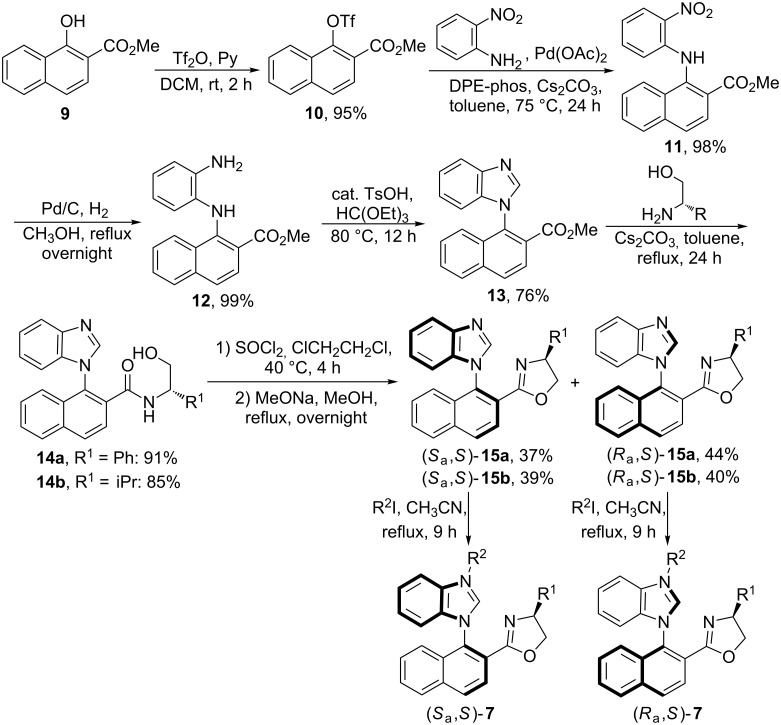
Synthesis of axially chiral benzimidazole derivatives.

Initially, we attempted to synthesize the desired axially chiral ligands **7**, and the synthetic route is shown in Scheme 1. Using methyl 1-hydroxy-2-naphthoate (**9**) as the starting material, trifluoromethylation with Tf_2_O in the presence of pyridine afforded its trifluoromethanesulfonate **10** in 95% yield, which was made to react with 2-nitroaniline in the presence of Pd(OAc)_2_/DPE-phos catalytic system to give the corresponding coupling compound **11** in 98% yield (Scheme 1). Subsequent reduction of compound **11** with Pd/C under a hydrogen atmosphere produced the desired compound **12** in 99% yield. Cyclization of **12** in the presence of triethyl orthoformate and a catalytic amount of TsOH at 80 °C gave the corresponding benzimidazole derivative **13** in 76% yield, which was further treated with (*S*)-2-amino-2-phenylethanol in the presence of Cs_2_CO_3_ in toluene to afford the corresponding amide **14a** as a mixture of diastereomeric isomers successfully in 91% yield. To our delight, the two diastereomeric isomers with a chiral *N*-naphthyl axis, (*S*_a_,*S*)-**15a** and (*R*_a_,*S*)-**15a**, were synthesized from amide **14a** according to the classical synthetic method for the preparation of an oxazoline ring, and were easily isolated by silica gel column chromatography in 37% yield and 44% yield, respectively. Similarly, using L-valinol, (*S*_a_,*S*)-**15b** and (*R*_a_,*S*)-**15b** could also be synthesized. Quaternization of the benzimidazole ring of (*S*_a_,*S*)-**15** and (*R*_a_,*S*)-**15** with R^2^I (R^2^ = Me, Et) gave the corresponding benzimidazolium salts **7**, respectively.

### Coordination study with AuCl·SMe_2_

**Figure 3:**
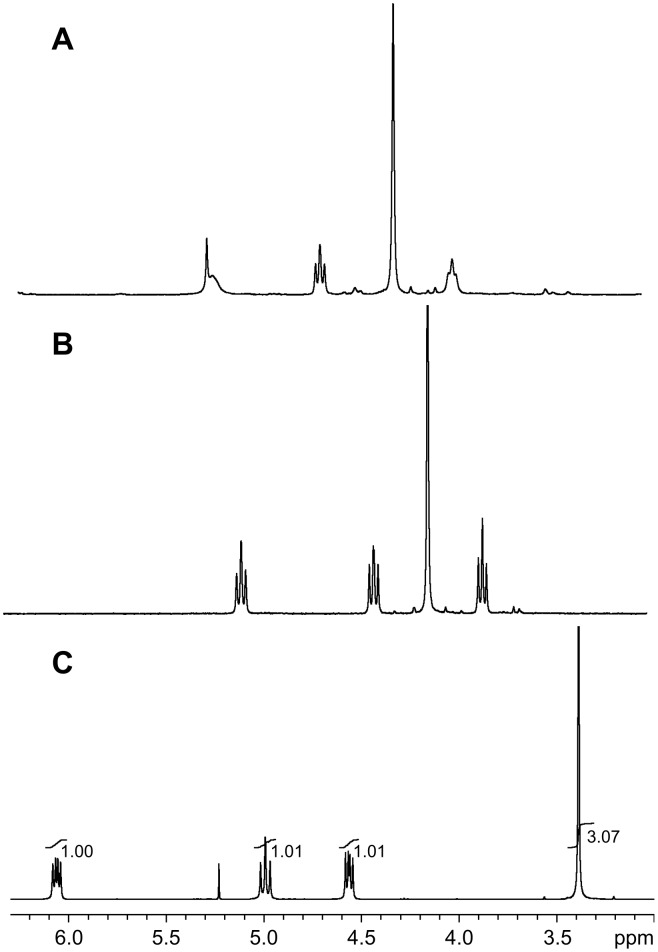
The coordination study of the NHC precursor (*S*_a_,*S*)-**7aa** with metal salts by ^1^H NMR analysis of the chemical shifts of protons on the oxazoline ring. (A) The ^1^H NMR spectrum of NHC precursor (*S*_a_,*S*)-**7aa**. (B) The ^1^H NMR spectrum of Au(I)-complex (*S*_a_,*S*)-**16aa**. (C) The ^1^H NMR spectrum of Pd(II)-complex (*S*_a_,*S*)-**8** (R^2^ = Me).

**Figure 4:**
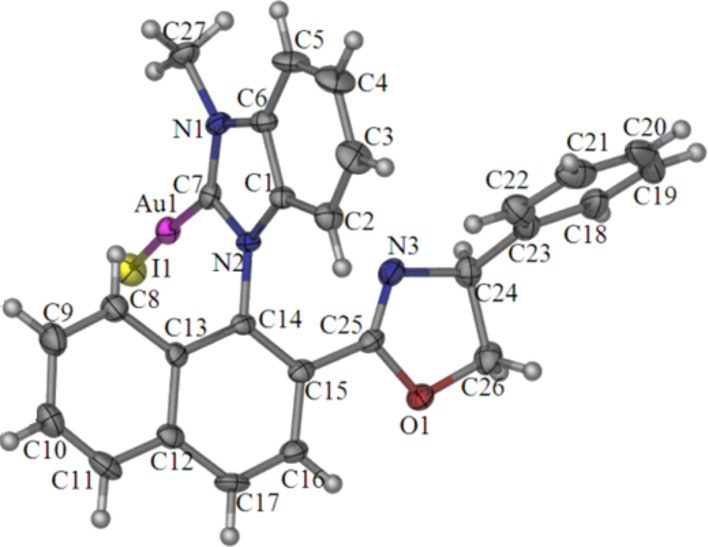
Solid-state molecular structure of (*S*_a_,*S*)-**16aa** with thermal ellipsoids at the 30% probability level. Selected bond distances (Å) and angles (°): Au1–I1 2.5260(5), Au1–C7 1.991(5), C7–N1 1.334(7), C7–N2 1.357(7), N2–C14 1.427(6), I1–Au1–C7 173.12(16), Au1–C7–N2 124.5(4), C7–N2–C14–C15 105.8(6).

The coordination behavior of ligand **7** with Pd(OAc)_2_ has been disclosed in previous work. However, only (*S*_a_,*S*)-**7** could give the corresponding Pd-complex (*S*_a_,*S*)-**8**, while the Pd(II)-complex with *R*-geometry of the chiral *N*-naphthyl axis could not be obtained from (*R*_a_,*S*)-**7**. With the NHC precursors **7** in hand, their coordination with AuCl·SMe_2_ in the presence of NaOAc in acetonitrile was further examined. Au(I)-complexes were isolated by flash column chromatography. Comparing the chemical shifts of protons on the oxazoline ring of the Au(I)-complex (*S*_a_,*S*)-**16aa** (R^1^ = Ph, R^2^ = Me) in the ^1^H NMR spectrum (Figure 3B) with those of the NHC precursor (*S*_a_,*S*)-**7aa** (Figure 3A), we found that the chemical shifts of these protons did not change much, suggesting that the chiral oxazoline group may not coordinate with the Au atom. However, according to the analysis of the ^1^H NMR spectrum of the Pd(II)-complex (*S*_a_,*S*)-**8** (R^2^ = Me) (Figure 3C), the chemical shifts of the protons of the coordinated oxazoline group changed significantly. In order to confirm this hypothesis, complex (*S*_a_,*S*)-**16aa** was recrystallized from CH_2_Cl_2_/petroleum ether (1/3, v/v), and its structure was established by single-crystal X-ray diffraction studies (Figure 4; Supporting Information File 1). The crystal data of Au(1)–C(7) (1.991(5) Å) is a typical Au–C^NHC^ bond length, in-line with those of other reported examples [16–21]. Furthermore, the angle of I(1)–Au(1)–C(7) = 173.12(16)° suggests a nearly linear coordination geometry around the gold(I) center, which is also a typical feature for known gold(I) complexes. Moreover, no coordinated oxazoline group in the complexes (*S*_a_,*S*)-**16** and (*R*_a_,*S*)-**16** could be further coordinated with other metal atoms such as Pd and Cu to furnish dual-metal catalysts, which is a hot research field in asymmetric catalysis [22–24].

Au(I)-complexes (*S*_a_,*S*)-**16** with different *N*-substituents or groups on the oxazoline ring were synthesized from the corresponding NHC precursors **7**. The yields are summarized in Table 1. It was found that the geometry the of chiral *N*-naphthyl axis had a significant influence on the yields of the Au(I)-complexes. The salts (*S*_a_,*S*)-**7** with an *S*-geometry of the chiral *N*-naphthyl axis gave higher yields of the corresponding Au(I)-complexes than did the salts (*R*_a_,*S*)-**7**, presumably due to the steric repulsion of the phenyl group on the oxazoline ring [25–29]. For example, salt (*S*_a_,*S*)-**7aa** gave the corresponding complex (*S*_a_,*S*)-**16aa** in up to 95% yield (Table 1, entry 1), while salt (*R*_a_,*S*)-**7aa** gave the corresponding complex (*R*_a_,*S*)-**16aa** in only 50% yield (Table 1, entry 2).

**Table 1:** The coordination study with AuCl·SMe_2_.

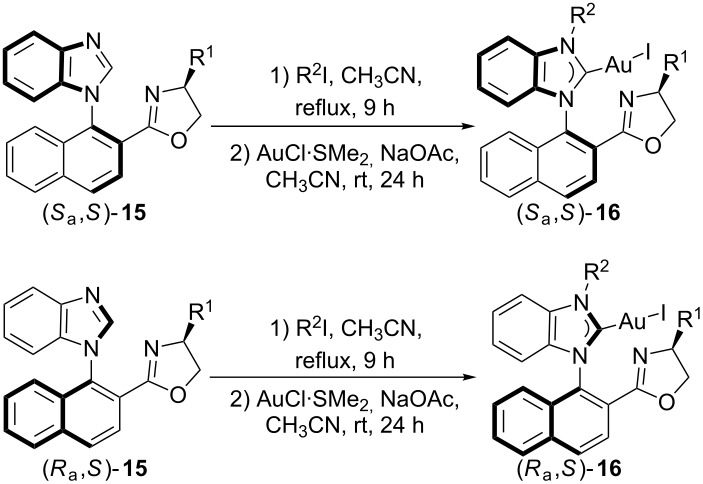					

entry	compound **15**	R^2^I	salt **7**	Au(I)-complex	yield (%)^a^

1	(*S*_a_,*S*)-**15a** (R^1^ = Ph)	MeI	(*S*_a_,*S*)-**7aa**	(*S*_a_,*S*)-**16aa**	95
2	(*R*_a_,*S*)-**15a** (R^1^ = Ph)	MeI	(*R*_a_,*S*)-**7aa**	(*R*_a_,*S*)-**16aa**	50
3	(*S*_a_,*S*)-**15a** (R^1^ = Ph)	EtI	(*S*_a_,*S*)-**7ab**	(*S*_a_,*S*)-**16ab**	89
4	(*R*_a_,*S*)-**15a** (R^1^ = Ph)	EtI	(*R*_a_,*S*)-**7ab**	(*R*_a_,*S*)-**16ab**	48
5	(*S*_a_,*S*)-**15b** (R^1^ = iPr)	MeI	(*S*_a_,*S*)-**7ba**	(*S*_a_,*S*)-**16ba**	90
6	(*R*_a_,*S*)-**15b** (R^1^ = iPr)	MeI	(*R*_a_,*S*)-**7ba**	(*R*_a_,*S*)-**16ba**	56

^a^isolated yield.

## Conclusion

NHC-oxazoline bidentate ligands with an axially chiral *N*-naphthyl framework were synthesized, and their coordination manners with AuCl·SMe_2_ were investigated. Diastereomeric NHC precursors **7**, (*S*_a_,*S*)-**7** and (*R*_a_,*S*)-**7**, gave the corresponding axially chiral NHC–Au complexes bearing a noncoordinated oxazoline group. However, while the ligands (*S*_a_,*S*)-**7** gave their corresponding Au complexes (*S*_a_,*S*)-**16** in excellent yields, only modest yields of complexes (*R*_a_,*S*)-**16** were obtained for ligands (*R*_a_,*S*)-**7**. Further studies focusing on the preparation of conformationally stable and enantiomerically pure transition-metal catalysts with an axially chiral *N*-aryl framework are currently in progress, and their applications in asymmetric catalysis are also undergoing.

## Supporting Information

File 1 Experimental procedures and characterization data of compounds.

File 2 Crystallographic data of (*S*_a_,*S*)-**16aa**. These data (CCDC 785035) can be obtained free of charge from the Cambridge Crystallographic Data Centre via http://www.ccdc.cam.ac.uk/data_request/cif.
Crystal structure data for NHC–Au(I) complex **16aa**.
