# Supplementary material for: Synthesis of axially chiral oxazoline–carbene ligands with an N-naphthyl framework and a study of their coordination with AuCl·SMe2
Source: Beilstein J Org Chem. 2012 May 11;8:726–31. doi: 10.3762/bjoc.8.81 (PMC3388859; doi:10.3762/bjoc.8.81)
Supplement: File 1 — Experimental procedures and characterization data of compounds. [file Beilstein_J_Org_Chem-08-726-s001.pdf]

Supporting Information

for

Synthesis of axially chiral oxazoline–carbene ligands with an *N*-naphthyl framework and a study of their coordination with $AuCl·SMe_2$

Feijun Wang*[1], Shengke Li[1], Mingliang Qu[1], Mei-Xin Zhao[1], Lian-Jun Liu[1] and Min Shi*[1,2]

Address: [1]Key Laboratory for Advanced Materials and Institute of Fine Chemicals, East China University of Science and Technology, and 130 MeiLong Road, Shanghai 200237, P. R. China and [2]State Key Laboratory of Organometallic Chemistry, Shanghai Institute of Organic Chemistry, Chinese Academy of Sciences, 354 Fenglin Road, Shanghai 200032, P. R. China.

Email: Feijun Wang - feijunwang@ecust.edu.cn; Min Shi - Mshi@mail.sioc.ac.cn

*Corresponding author

Experimental procedures and characterization data of compounds

Contents

General remarks S2

Synthesis of axially chiral ligands (*S*a,*S*)-**15** and (*R*a,*S*)-**15** S3

General procedures for the synthesis of axially chiral Au(I) complexes (*S*a,*S*)-**16** and (*R*a,*S*)-**16** S14

References S21

General remarks: Dichloromethane was freshly distilled from calcium hydride; THF and toluene were distilled from sodium (Na) under an argon (Ar) atmosphere. Melting points were determined on a digital melting-point apparatus and temperatures were uncorrected. ^{1}H NMR, ^{19}F NMR and ^{13}C NMR spectra were recorded on Bruker AM-300 or AM-400 spectrophotometers. Infrared spectra were recorded on a Perkin-Elmer PE-983 spectrometer with absorption in cm^{-1}. Flash column chromatography was performed using 300–400 mesh silica gel. For thin-layer chromatography (TLC), silica gel plates (Huanghai GF_{254}) were used. Mass spectra were recorded by EI and ESI, and HRMS were measured on a HP-5989 instrument. Optical rotations were determined at 589 nm (sodium D line) by using a Perkin-Elmer-341 MC digital polarimeter; $[\alpha]_D$ values are given in units of 10 deg^{-1} cm^2 g^{-1}.

Synthesis of axially chiral ligands (*S*$_a$,*S*)-15 and (*R*$_a$,*S*)-15.

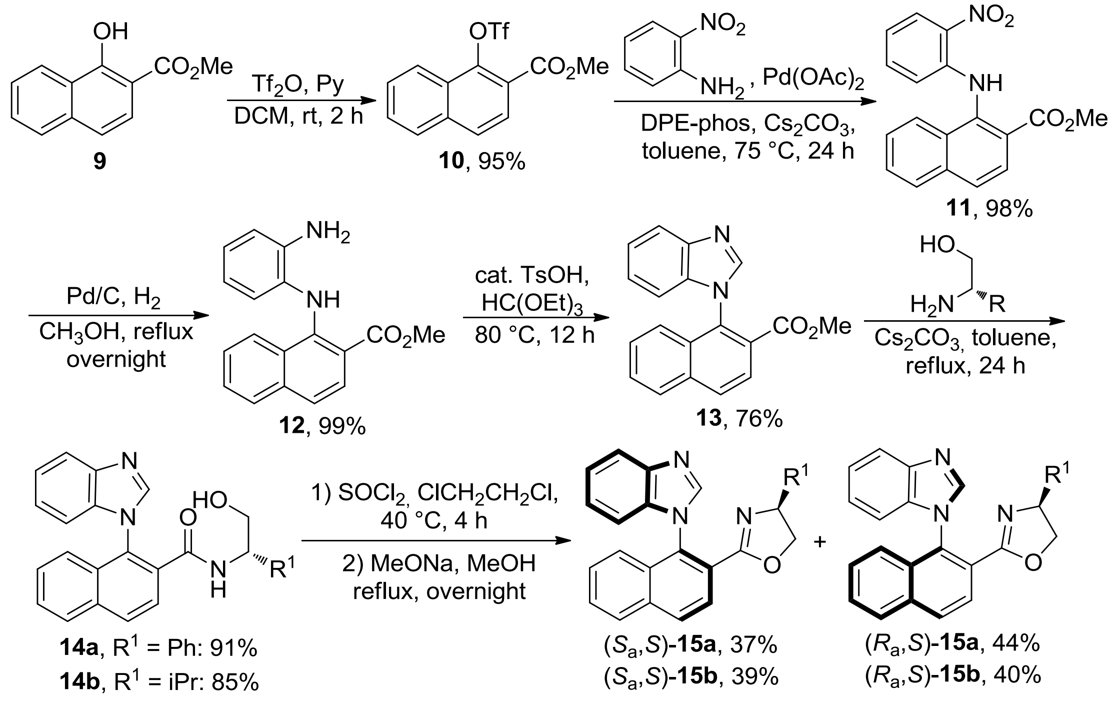

Methyl 1-(trifluoromethylsulfonyloxy)-2-naphthoate (**10**) [1]

Trifluoromethanesulfonic anhydride (1.1 mL, 6.5 mmol) was added dropwise to the solution of methyl 1-hydroxy-2-naphthoate (**9**) (1.01 g, 5.0 mmol) and pyridine (0.5 mL, 6.2 mol) in dried CH_2Cl_2 (12 mL) at 0 °C, and the resulting solution was stirred for 2 h at room temperature. The reaction mixture was diluted with CH_2Cl_2, and washed with water and saturated sodium bicarbonate solution. The organic layer was dried over $MgSO_4$. The solvent was removed under reduced pressure and the crude product was purified by flash chromatography (ethyl acetate/petroleum ether 1/16) to afford **10** as a light yellow solid in 95% yield (1.59 g, 4.75 mmol). ^{1}H NMR (400 MHz, $CDCl_3$) δ 4.02 (s, 3H), 7.67–7.70 (m, 2H), 7.90–7.94 (m, 2H), 8.00 (d, *J* = 8.8 Hz, 1H), 8.18–8.20 (m, 1H); ^{1}H NMR (400 MHz, $CDCl_3$) δ 52.8, 113.8, 117.0, 120.2, 121.4, 122.1, 123.4, 126.2, 128.0, 128.1, 128.2, 129.1, 136.6, 144.7, 165.3; ^{19}F NMR (470 MHz, $CDCl_3$) δ −73.0; MS (ESI) *m/z* (%): 333.0 [M – H] (100); HRMS (ESI) calcd. for $C_{13}H_8O_5SF_3$ 333.0045; found 333.0044.

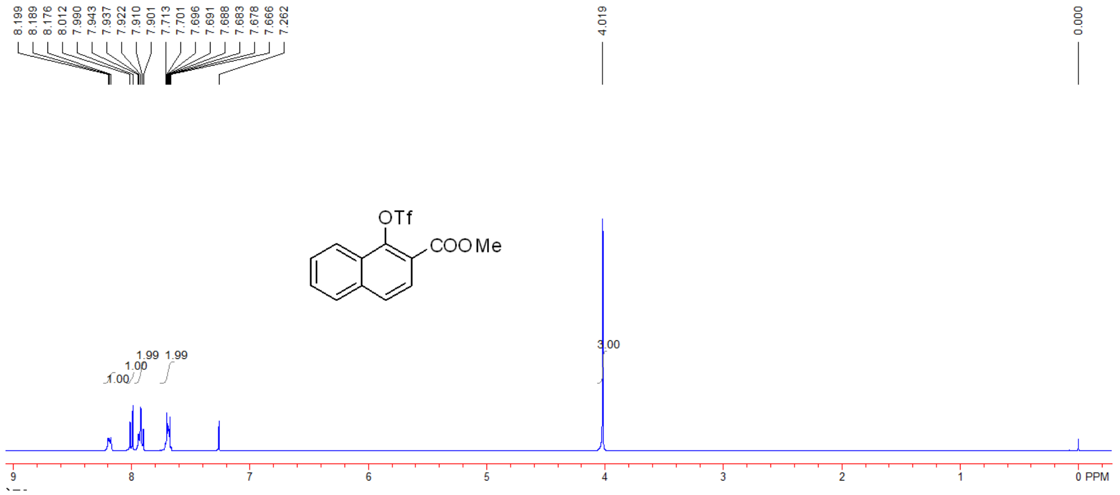

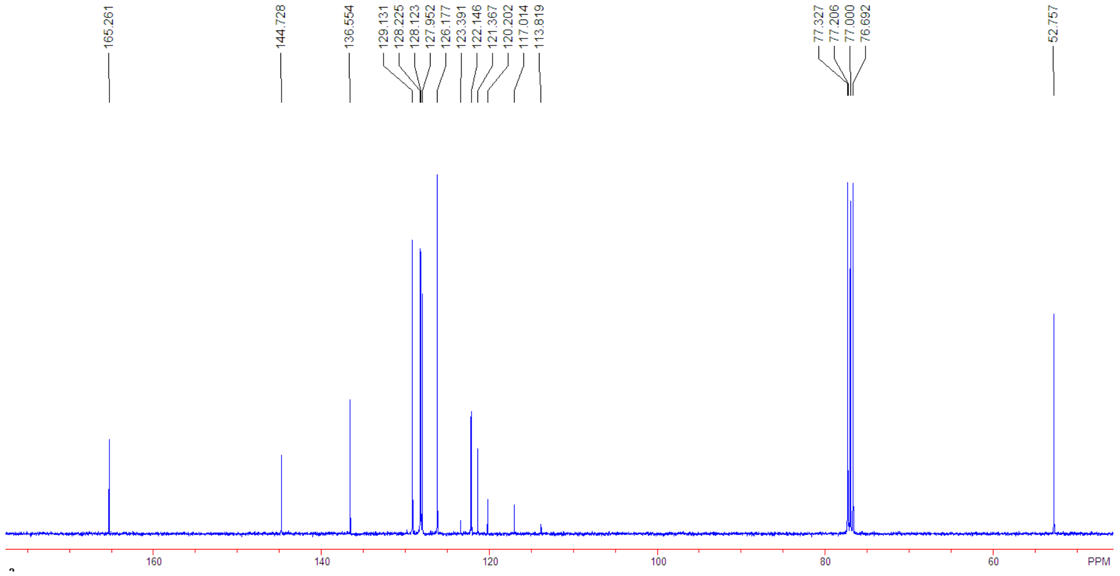

Methyl 1-(2-nitrophenylamino)-2-naphthoate (**11**)

Methyl 1-(trifluoromethylsulfonyloxy)-2-naphthoate **10** (0.67 g, 2.0 mmol), 2-nitroaniline (0.30 g, 2.2 mmol), $Pd(OAc)_2$ (22.5 mg, 0.1 mmol), DPE-phos (108.0 mg, 0.2 mmol), and Cs_2CO_3 (0.98 g, 3.0 mmol) were stirred in anhydrous toluene (10 mL) at 75 °C until the reaction was completed. The reaction mixture was cooled to room temperature, and was filtered by diatomite. The filtrate was washed with water, extracted with DCM (3 × 20 mL) and dried over anhydrous $MgSO_4$. The solvent was removed under reduced pressure and the residue was purified by silica gel flash column chromatography (ethyl acetate/petroleum ether 1/10) to afford the desired product **11** as an orange solid in 98% yield (0.63 g, 1.95 mmol); mp 141.6–142.9 °C; ^{1}H NMR (400 MHz, $CDCl_3$, TMS) δ 3.90 (s, 3H), 6.34 (d, *J* = 8.4 Hz, 1H), 6.78–6.82 (m, 1H), 7.15–7.19 (m, 1H), 7.43–7.47 (m, 1H), 7.58–7.62 (m, 1H), 7.81 (d, *J* = 8.4 Hz, 1H), 7.91 (t, *J* = 7.2 Hz, 2H), 8.05 (d, *J* = 8.4 Hz,

1H), 8.24 (dd, *J* = 1.6, 8.4 Hz, 1H), 10.48 (s, 1H); ^{13}C NMR (100 MHz, $CDCl_3$, TMS) δ 52.4, 117.4, 118.1, 122.5, 125.5, 126.2, 126.4, 127.0, 128.4, 128.5, 129.3, 134.4, 135.0, 136.1, 137.9, 143.3, 167.0; MS (ESI) *m/z* (%): 323.1 [M + H] (21); HRMS (ESI) calcd. for $C_{18}H_{15}N_2O_4$ 323.1032; found 323.1036.

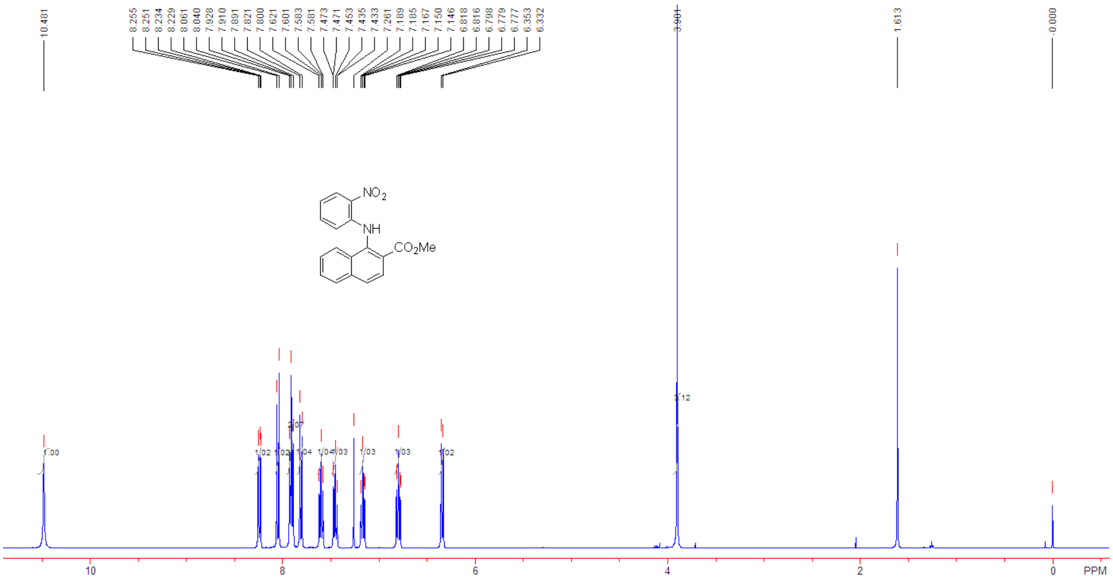

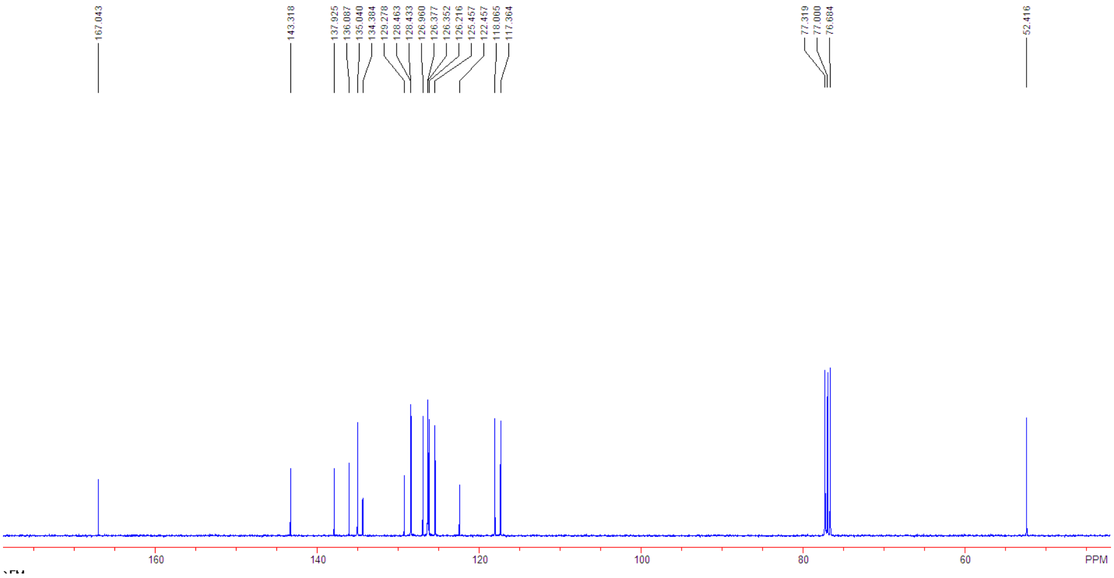

Methyl 1-(2-aminophenylamino)-2-naphthoate (**12**)

A mixture of **11** (0.97 g, 3.0 mmol), 10% Pd-C with 50% H_2O (0.32 g) in a solution of MeOH (14 mL) was stirred overnight under reflux under 1 atm of H_2. After cooling to room temperature, Pd–C was removed by filtration, and the resulting solution was evaporated to remove the solvent under reduced pressure. The residue was purified by silica gel flash column chromatography

(ethyl acetate/petroleum ether 1/10, 1% NEt_3 added) to afford the desired product **12** as a yellow solid in 99% yield (0.87 g, 2.98 mmol); mp 134.3–136.1 °C; ^{1}H NMR ($CDCl_3$, 400 MHz, TMS) δ 1.64 (br s, 1H), 3.93 (s, 3H), 4.06 (br s, 2H), 6.41 (d, *J* = 8.0 Hz, 1H), 6.47–6.51 (m, 1H), 6.81–6.89 (m, 2H), 7.20–7.25 (m, 1H), 7.41–7.48 (m, 2H), 7.77 (t, *J* = 8.0 Hz, 2H), 7.98 (d, *J* = 8.8 Hz, 1H), 9.23 (s, 1H); ^{13}C NMR ($CDCl_3$, 100 MHz, TMS) δ 52.0, 113.6, 115.6, 118.6, 121.0, 121.4, 123.8, 125.1, 125.9, 126.5, 126.7, 128.1, 128.2, 133.2, 136.8, 139.3, 147.6, 169.1; MS (ESI) *m/z* (%): 293.1 [M + H] (100); HRMS (ESI) calcd. for $C_{18}H_{17}N_2O_2$ 293.1290; found 293.1294.

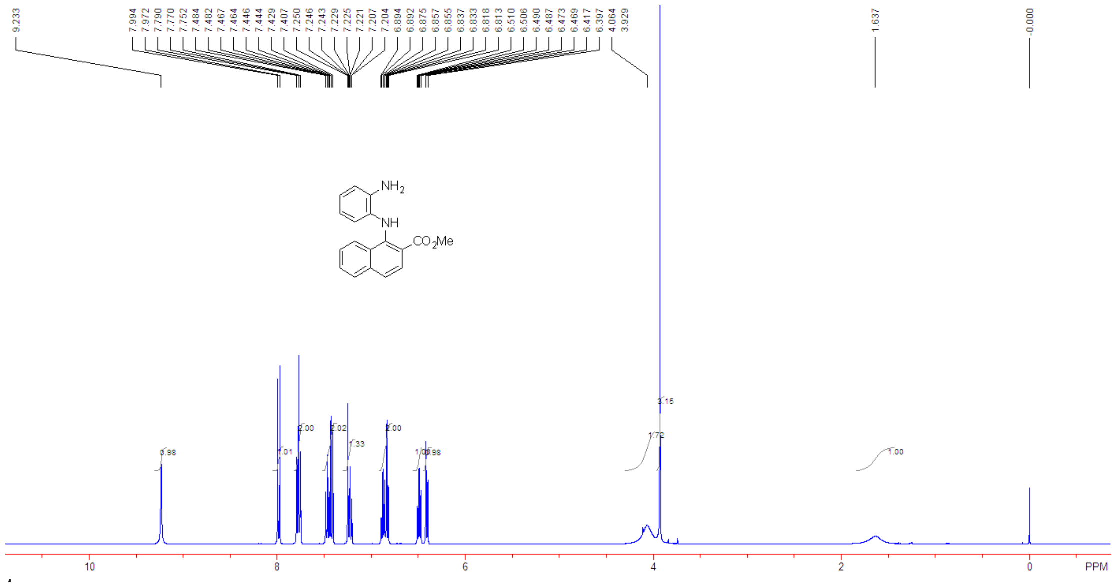

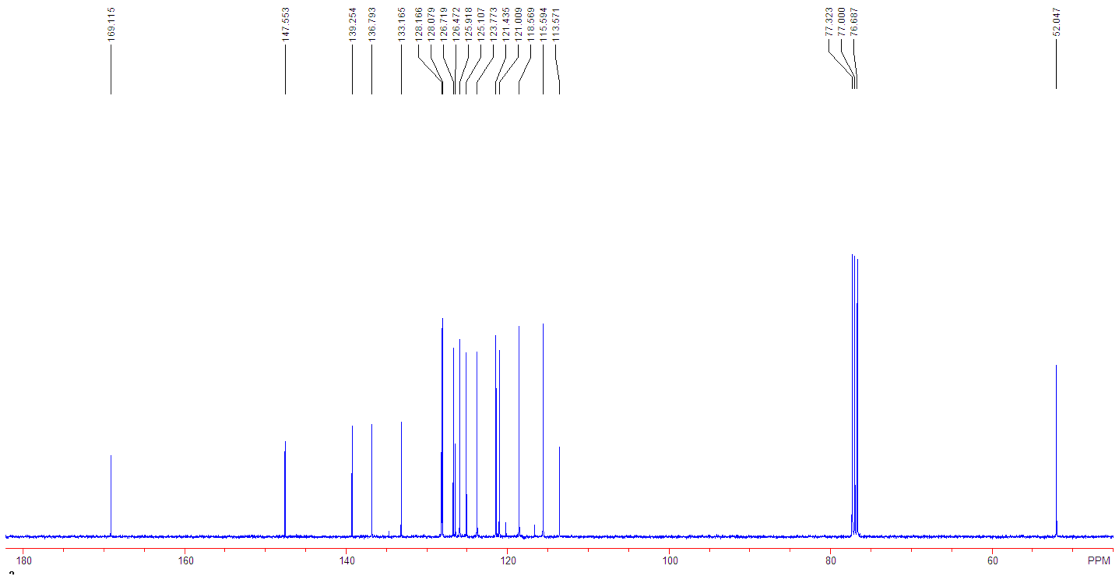

Methyl 1-(1*H*-benzo[d]imidazol-1-yl)-2-naphthoate (**13**)

Compound **12** (0.88 g, 3.0 mmol) and triethyl orthoformate [$HC(OC_2H_5)_3$] (8.0 mL) containing a catalytic amount of TsOH were heated at 115 °C until compound **12** was consumed. After cooling to room temperature, ethyl acetate was added to form an azeotropic mixture in order to remove the excess amount of triethyl orthoformate under reduced pressure. The residue was purified by silica gel flash column chromatography (ethyl acetate/petroleum ether 1/3) to afford the desired product **13** in 76% yield (0.69 g, 2.28 mmol). Viscous brown solid; mp 115.0–116.7 °C; ^{1}H NMR ($CDCl_3$, 400 MHz, TMS) δ 3.51 (s, 3H), 6.92 (d, *J* = 8.4 Hz, 1H), 7.22 (t, *J* = 8.0 Hz, 1H), 7.34 (t, *J* = 8.0 Hz, 2H), 7.49 (t, *J* = 8.0 Hz, 1H), 7.66 (t, *J* = 7.6 Hz, 1H), 7.95 (d, *J* = 8.0 Hz, 1H), 8.02 (d, *J* = 8.4 Hz, 1H), 8.04 (s, 1H), 8.11 (s, 2H); ^{13}C NMR ($CDCl_3$, 100 MHz, TMS) δ 52.5, 110.1, 120.3, 122.5, 123.6, 123.9, 126.1, 126.9, 128.2, 128.4, 128.8, 129.7, 130.7, 132.5, 135.7, 136.1, 143.0, 144.1, 165.8; MS (ESI) *m/z* (%): 303.1 [M + H] (100); HRMS (ESI) calcd. for $C_{19}H_{15}N_2O_2$ 303.1134; found 303.1134.

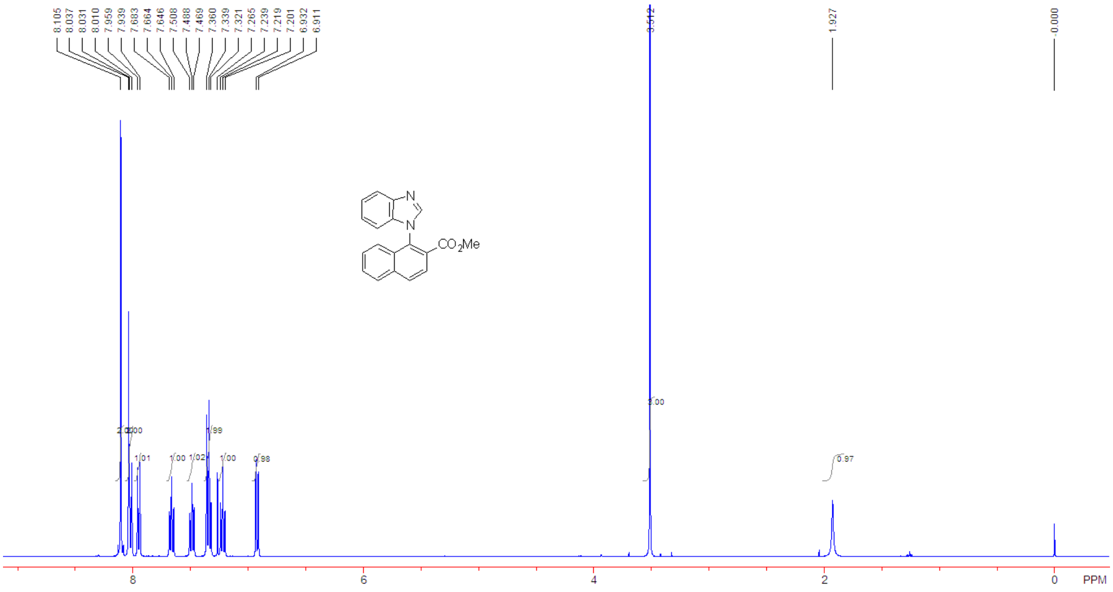

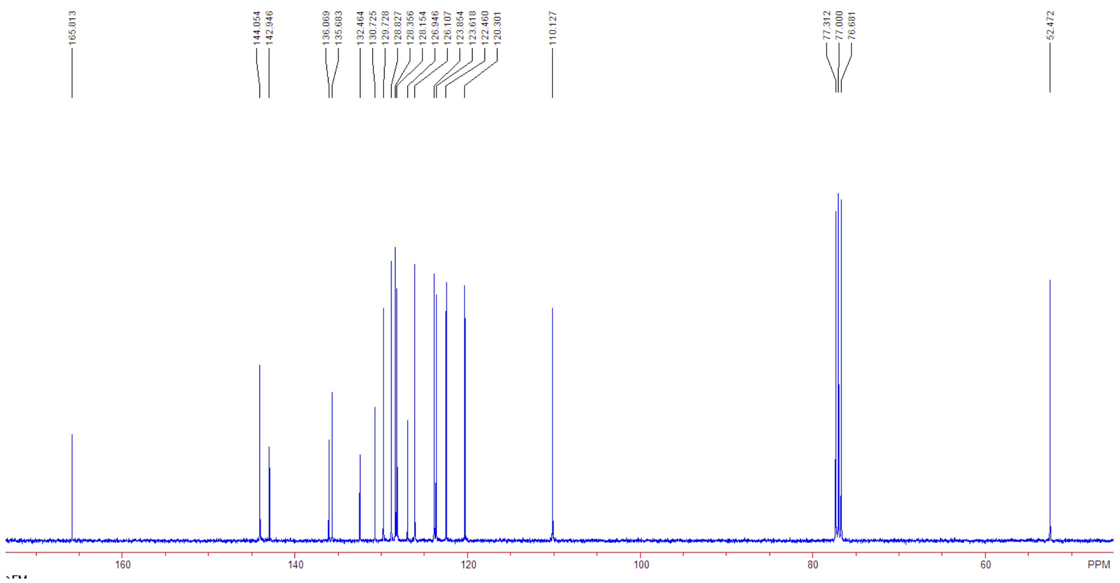

1-(1*H*-benzo[*d*]imidazol-1-yl)-*N*-((*S*)-2-hydroxy-1-phenylethyl)-2-naphthamide (**14a**)

A solution of (*S*)-2-amino-2-phenylethanol (1.10 g, 8.0 mmol), compound **13** (1.21 g, 4.0 mmol), Cs_2CO_3 (2.61 mg, 8.0 mmol), and toluene (20 mL) was stirred at 85 °C until compound **13** was consumed. After cooling to room temperature, the mixture was diluted with DCM, and washed with cold water and brine. The organic layer was dried over $MgSO_4$. After removal of the solvent in vacuo, the residue was purified by silica gel flash column chromatography (petroleum ether/ethyl acetate, 1/1 to 0/1) to afford the diastereomeric mixture **14a** in 91% yield (1.48 g, 3.63 mmol). Pale-red solid; mp 226.7–228.4 °C; ^{1}H NMR ($CDCl_3$, 400 MHz, TMS) δ 2.23 (br s, 2H), 3.31–3.37 (m, 1H), 3.40–3.48 (m, 1H), 4.86–4.90 (m, 1H), 6.00 (d, *J* = 7.2 Hz, 0.51H), 6.55 (d, *J* = 7.6 Hz, 0.46H), 6.65–6.67 (m, 1H), 6.82–6.85 (m, 1H), 7.01 (d, *J* = 8.0 Hz, 0.54H), 7.04 (d, *J* = 8.0 Hz, 0.50H), 7.08–7.15 (m, 3H), 7.19–7.25 (m, 1H), 7.33 (t, *J* = 7.2 Hz, 0.52H), 7.39 (t, *J* = 7.6 Hz, 0.56H), 7.46 (d, *J* = 7.6 Hz, 0.50H), 7.50 (d, *J* = 8.0 Hz, 0.53H), 7.61–7.65 (m, 1H), 7.82–7.89 (m, 2H), 7.97–8.09 (m, 3H); ^{13}C NMR ($CDCl_3$, 100 MHz, TMS) δ 55.7, 55.9, 65.4, 65.6, 110.4, 110.5, 120.5, 120.8, 123.2, 123.23, 123.26, 123.5, 124.3, 124.6, 125.3, 125.5, 126.32, 126.35, 127.57, 127.63, 128.2, 128.29, 128.36, 128.39, 128.46, 128.50, 128.63, 128.73, 128.84, 130.1, 130.22, 130.24, 130.41, 132.83, 132.86, 134.80, 134.84, 135.6, 135.8, 138.2, 142.8, 143.1, 143.9, 144.4, 166.5, 166.3; MS (ESI) *m/z* (%): 408.2 [M + H] (100); HRMS (ESI) calcd. for $C_{26}H_{22}N_3O_2$ 408.1712; found 408.1715.

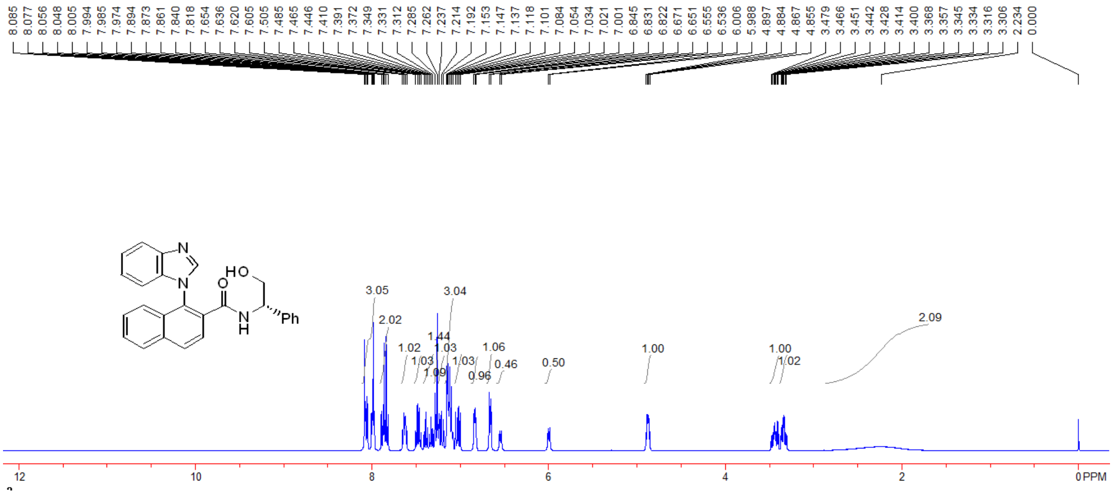

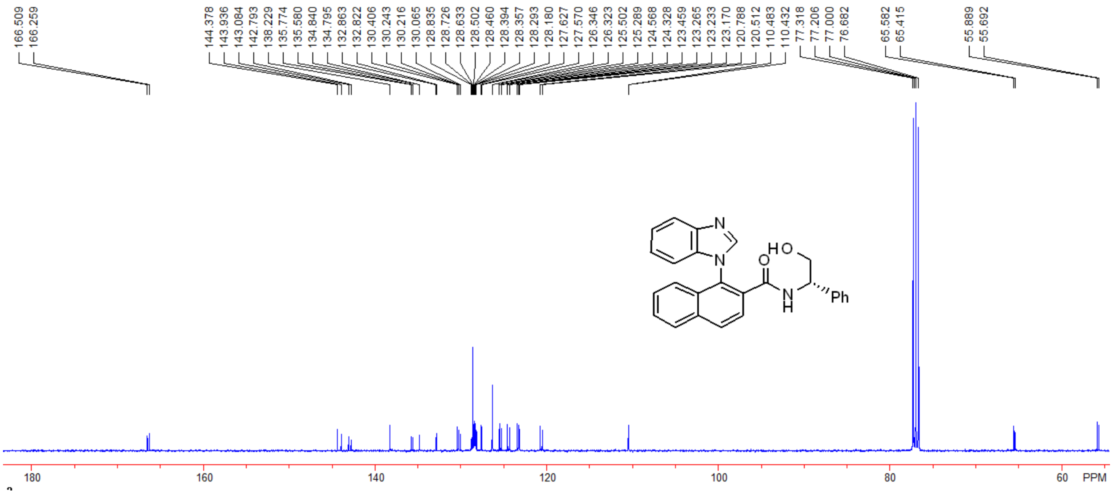

1-(1*H*-benzo[*d*]imidazol-1-yl)-*N*-((*S*)-1-hydroxy-3-methylbutan-2-yl)-2-naphthamide (**14b**)

The procedure to prepare **14b** using L-valinol as chiral amino alcohol was according to that of **14a**. Pale-red solid; mp 201.3–201.8 °C; ^{1}H NMR ($CDCl_3$, 400 MHz, TMS) δ 0.55 (d, *J* = 6.8 Hz, 1.5H), 058 (d, *J* = 6.8 Hz, 1.5H), 0.61 (d, *J* = 6.8 Hz, 1.5H), 0.68 (d, *J* = 6.8 Hz, 1.5H), 1.47–1.53 (m, 1H), 3.07 (dd, *J* = 3.6, 11.2 Hz, 0.5H), 3.13 (dd, *J* = 3.2, 10.8 Hz, 0.5H), 3.20 (dd, *J* = 4.8, 11.2 Hz, 0.5H), 3.33 (dd, *J* = 5.2, 11.2 Hz, 0.5H), 3.63–3.66 (m, 1H), 5.43 (d, *J* = 8.4 Hz, 0.5H), 5.82 (d, *J* = 8.8 Hz, 0.5H), 7.06 (dd, *J* = 3.2, 8.0 Hz, 1H), 7.22–7.32 (m, 2H), 7.35-7.41 (m, 2H), 7.49 (dd, *J* = 3.2, 11.2 Hz, 1H), 7.64 (dd, *J* = 3.2, 14.4 Hz, 1H), 7.87 (dd, *J* = 4.8, 8.4 Hz, 1H), 7.91 (d, *J* = 7.6 Hz, 0.5H), 7.96 (d, *J* = 8.4 Hz, 0.5H), 8.01 (t, *J* = 8.0 Hz, 1H), 8.08–8.13 (m, 2H); MS (ESI) *m/z* (%): 374.2 [M + H] (100); HRMS (ESI) calcd. for $C_{23}H_{24}N_3O_2$ 374.1869; found 374.1875.

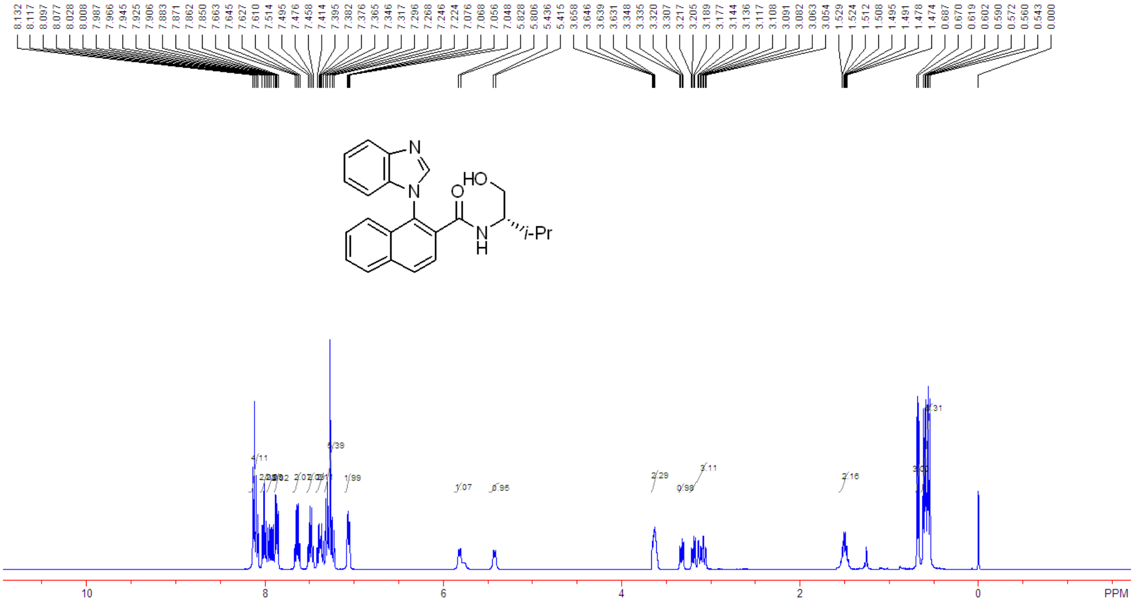

(S)-2-((S)-1-(1H-benzo[d]imidazol-1-yl)naphthalen-2-yl)-4-phenyl-4,5-dihydro oxazole ((S_a,S)-**15a**) and (S)-2-((R)-1-(1H-benzo[d]imidazol-1-yl)naphthalen-2-yl)-4-phenyl-4,5-dihydro oxazole ((R_a,S)-**15a**)

$SOCl_2$ (0.36 mL, 5.1 mol) was slowly added to a solution of diastereomeric mixture **14** (0.41 g, 1.0 mol) in dry 1,2-dichloroethane (10 mL) at 0 °C. The resulting solution was stirred at 40 °C for 4 h, and then the solvent was removed under reduced pressure. Subsequently, the residue was treated with sodium methoxide (0.43 g, 8.0 mol) in CH_3OH (15 mL), and stirred overnight under reflux. The resulting mixture was diluted with cold water, and extracted with DCM **(**3 × 20 mL). The organic layer was washed with brine, and dried over Na_2SO_4. After removal of the solvent under reduced pressure, the residue was purified by silica gel flash column chromatography (ethyl acetate/petroleum ether 1/2) to afford (S_a,S)-**15a** and (R_a,S)-**15a**.

(S_a,S)-**15a**: White solid, 37% yield; mp 186.1–187.9 °C; $[\alpha]^{20}_{D}$ +112.1 (c 0.70, $CHCl_3$); ^1H NMR ($CDCl_3$, 400 MHz, TMS) δ 3.90 (t, J = 8.8 Hz, 1H), 4.29 (dd, J = 8.4, 10.4 Hz, 1H), 5.15 (dd, J = 8.8, 10.4 Hz, 1H), 6.85–6.87 (m, 2H), 6.99 (d, J = 8.0 Hz, 1H), 7.19–7.24 (m, 4H), 7.34–7.38 (m, 2H), 7.47–7.51 (m, 1H), 7.63–7.67 (m, 1H), 7.94 (d, J = 8.0 Hz, 1H), 8.02 (d, J = 8.0 Hz, 1H), 8.10 (d, J = 8.4 Hz, 2H), 8.17 (d, J = 8.8 Hz, 1H); ^{13}C NMR ($CDCl_3$, 100 MHz, TMS) δ 69.7, 74.8, 110.3, 120.3, 122.5, 123.57, 123.58, 124.6, 126.3, 126.5, 127.4, 128.25, 128.32, 128.4, 128.6, 129.7, 130.9, 131.7, 135.3, 136.0, 141.5, 143.2, 144.4, 162.9; MS (ESI) m/z (%) 390.2 [M + H] (71); HRMS (ESI) calcd. for $C_{16}H_{20}N_3O$ 390.1606; found 390.1611.

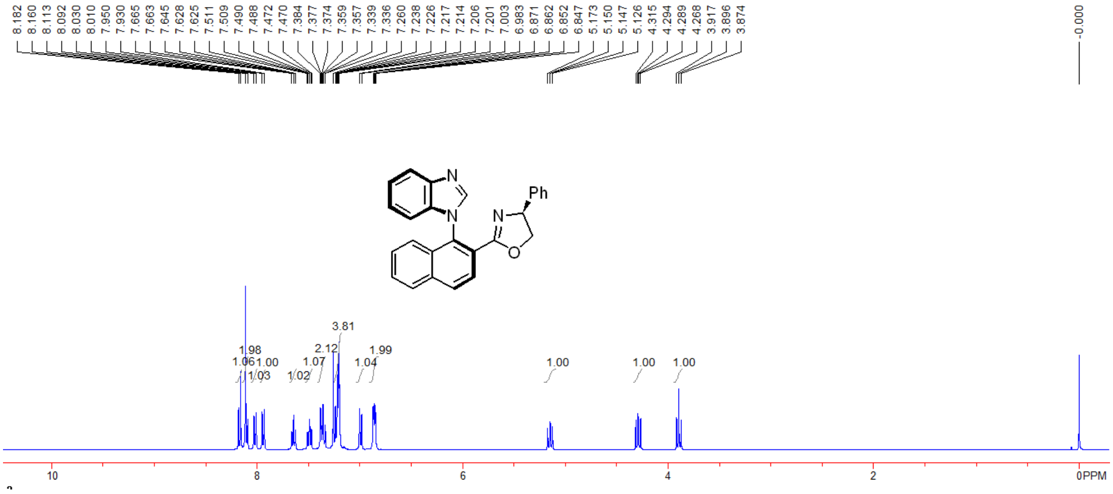

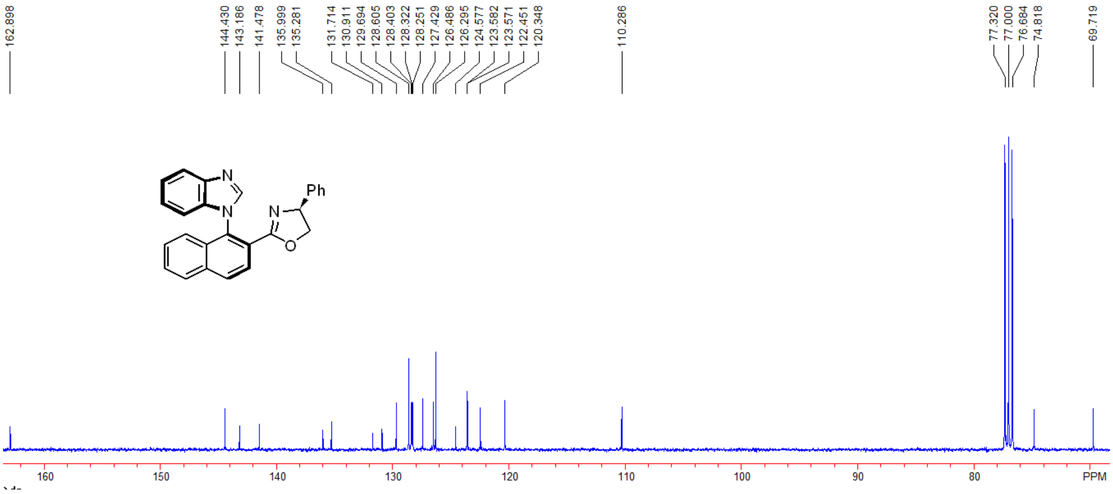

(R_a,S)-**15a**: White solid, 44% yield; mp 153.6–155.4 °C; $[\alpha]^{20}_{D}$ −62.5 (*c* 0.80, $CHCl_3$); ^{1}H NMR ($CDCl_3$, 400 MHz, TMS) δ 3.64 (t, *J* = 8.8 Hz, 1H), 4.43 (dd, *J* = 8.8, 10.0 Hz, 1H), 5.14 (dd, *J* = 9.2, 10.4 Hz, 1H), 6.92–6.94 (m, 2H), 7.02 (d, *J* = 8.0 Hz, 1H), 7.21–7.25 (m, 4H), 7.33–7.37 (m, 1H), 7.39 (d, *J* = 8.8 Hz, 1H), 7.47–7.52 (m, 1H), 7.62–7.67 (m, 1H), 7.94 (d, *J* = 8.4 Hz, 1H), 8.02 (d, *J* = 8.4 Hz, 1H), 8.10 (d, *J* = 10.4 Hz, 2H), 8.15 (d, *J* = 8.8 Hz, 1H); ^{13}C NMR ($CDCl_3$, 100 MHz, TMS) δ 69.8, 75.2, 110.4, 120.3, 122.5, 123.5, 123.6, 124.9, 126.6, 126.7, 127.6, 128.28, 128.32, 128.4, 128.6, 129.7, 130.8, 131.7, 135.3, 136.1, 141.3, 143.1, 144.4, 163.5; MS (ESI) *m/z* (%): 390.2 [M + H] (71); HRMS (ESI) calcd. for $C_{16}H_{20}N_3O$ 390.1606; found 390.1611.

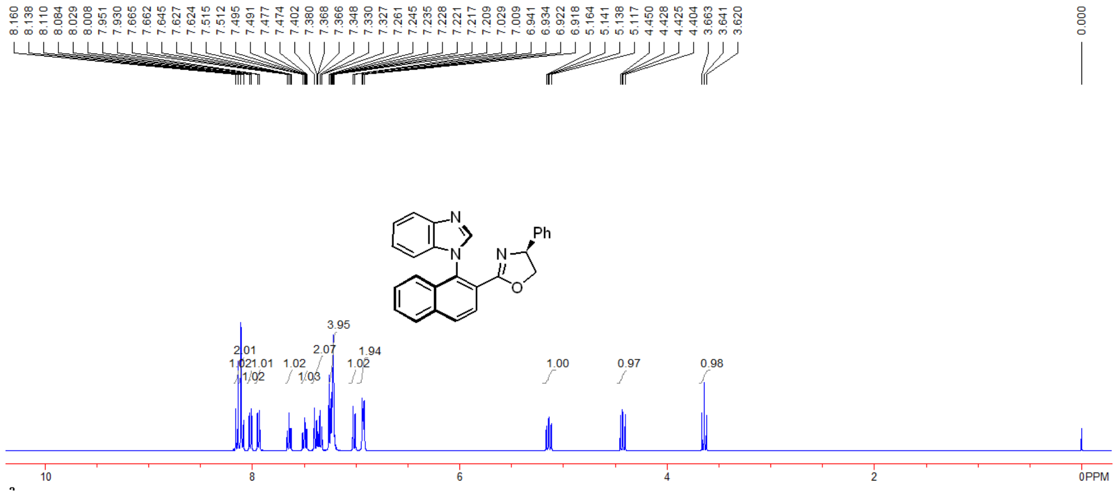

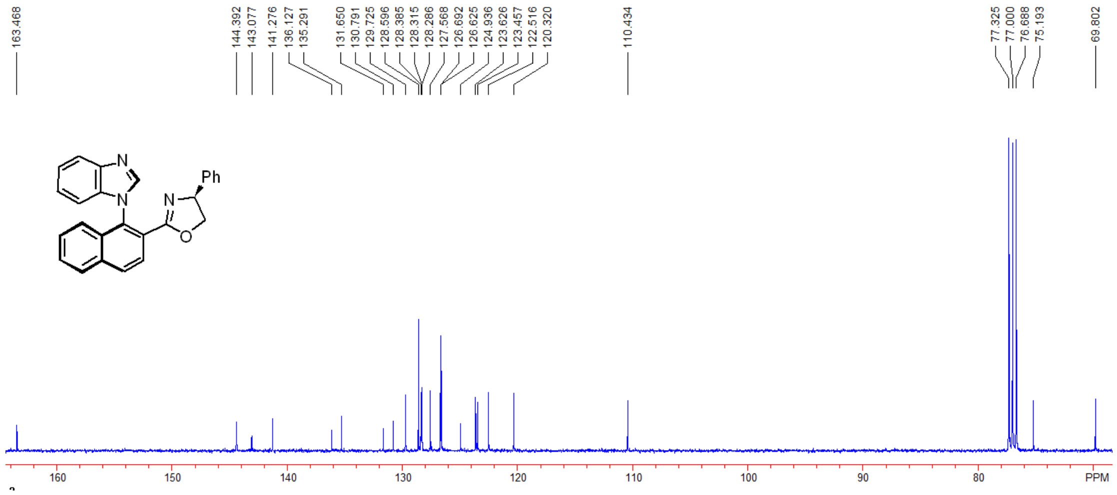

(*S*)-2-((*S*)-1-(1*H*-benzo[*d*]imidazol-1-yl)naphthalen-2-yl)-4-isopropyl-4,5-dihydrooxazole ((*S*$_a$,*S*)-**15b**) and (*S*)-2-((*R*)-1-(1*H*-benzo[*d*]imidazol-1-yl)naphthalen-2-yl)-4-isopropyl-4,5-dihydrooxazole ((*R*$_a$,*S*)-**15b**)

The procedure to prepare **15b** was according to that of **15a**.

(*S*$_a$,*S*)-**15b**. White solid, 39% yield; mp 151.0–151.5 °C; $[\alpha]^{20}_{D}$ −101 (*c* 0.80, $CHCl_3$); ^1H NMR ($CDCl_3$, 400 MHz, TMS) δ 0.698 (d, *J* = 7.2 Hz, 3H), 0.701 (d, *J* = 7.2 Hz, 3H), 1.38–1.43 (m, 1H), 3.74 (t, *J* = 8.0 Hz, 1H), 3.79–3.84 (m, 1H), 3.92 (dd, *J* = 7.2, 9.2 Hz, 1H), 6.93 (d, *J* = 8.0 Hz, 1H), 7.19 (t, *J* = 8.0 Hz, 1H), 7.31 (dd, *J* = 7.6, 8.0 Hz, 1H), 7.37 (d, *J* = 8.8 Hz, 1H), 7.47 (d, *J* = 8.0 Hz, 1H), 7.62 (dd, *J* = 7.2, 8.0 Hz, 1H), 7.92 (d, *J* = 8.0 Hz, 1H), 8.00 (d, *J* = 8.0 Hz, 1H), 8.07 (s, 3H); ^{13}C NMR ($CDCl_3$, 100 MHz, TMS) δ 18.2, 18.3, 32.6, 70.6, 72.6, 110.3, 120.2, 122.3, 123.4, 123.5, 124.9, 126.4, 128.19, 128.21, 129.6, 130.8, 131.4, 135.1, 135.9, 143.1, 144.5, 161.4; MS (ESI) *m/z* (%): 356.2 [M + H] (71); HRMS (ESI) calcd. for $C_{23}H_{22}N_3O$ 356.1763; found 356.1762.

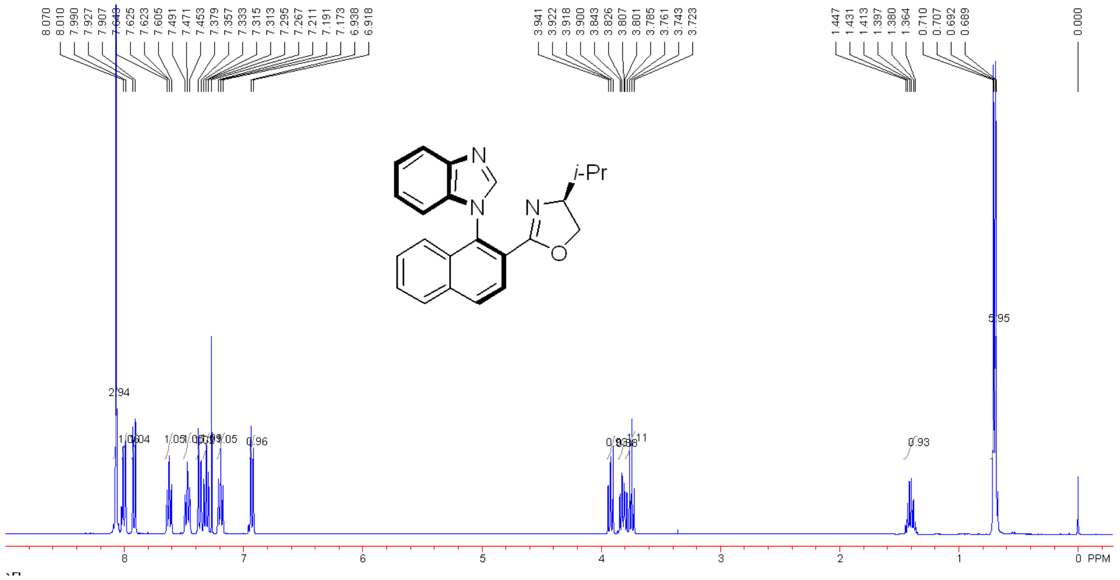

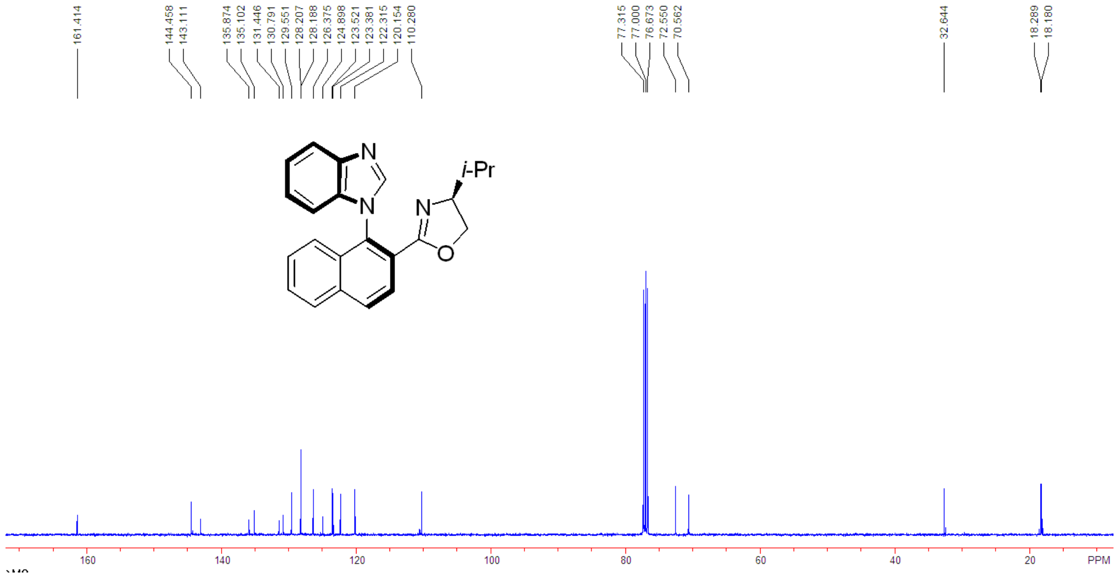

(R_a,S)-**15b**. White solid, 40% yield; mp 138.4–139.0 °C; $[\alpha]^{20}_{D}$ −42 (*c* 0.50, $CHCl_3$); ^1H NMR ($CDCl_3$, 400 MHz, TMS) δ 0.68 (d, *J* = 6.4 Hz, 3H), 0.76 (d, *J* = 6.4 Hz, 3H), 1.50–1.58 (m, 1H), 3.57 (t, *J* = 8.4 Hz, 1H), 3.80–3.86 (m, 1H), 4.07 (t, *J* = 8.4 Hz, 1H), 6.95 (d, *J* = 7.6 Hz, 1H), 7.20 (t, *J* = 7.6 Hz, 1H), 7.30–7.35 (m, 2H), 7.47 (t, *J* = 7.2 Hz, 1H), 7.62 (t, *J* = 7.2 Hz, 1H), 7.92 (d, *J* = 8.0 Hz, 1H), 7.99–8.07 (m, 4H); ^{13}C NMR ($CDCl_3$, 100 MHz, TMS) δ 18.0, 18.6, 70.7, 72.5, 110.6, 120.1, 122.4, 123.4, 123.48, 123.52, 125.3, 126.5, 128.17, 128.23, 129.6, 130.7, 131.4, 135.1, 136.1, 143.0, 144.3, 161.8; MS (ESI) *m/z* (%): 356.2 [M + H] (71); HRMS (ESI) calcd. for $C_{23}H_{22}N_3O$ 356.1763; found 356.1762.

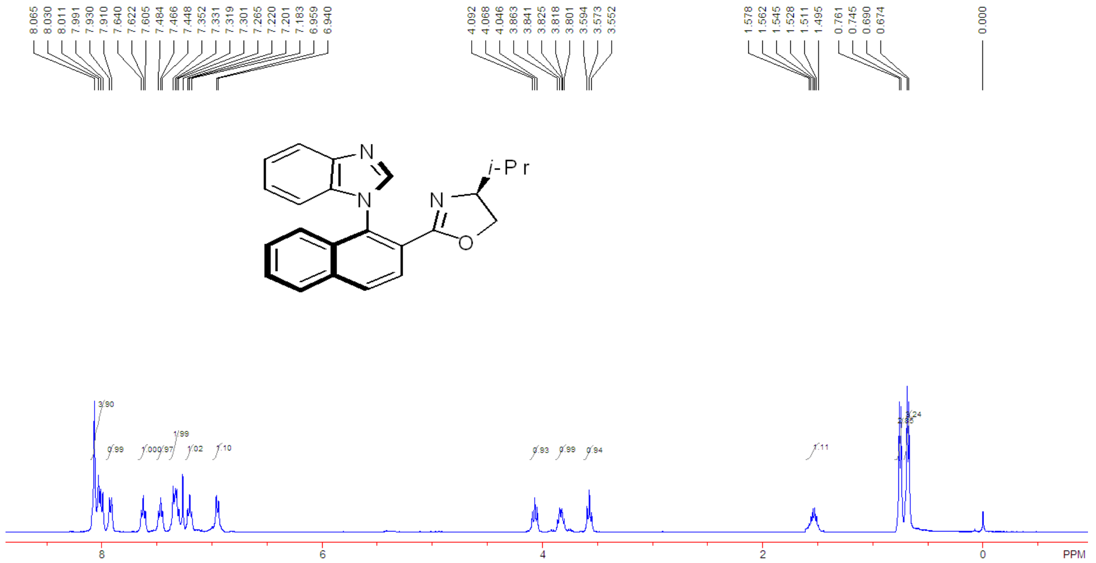

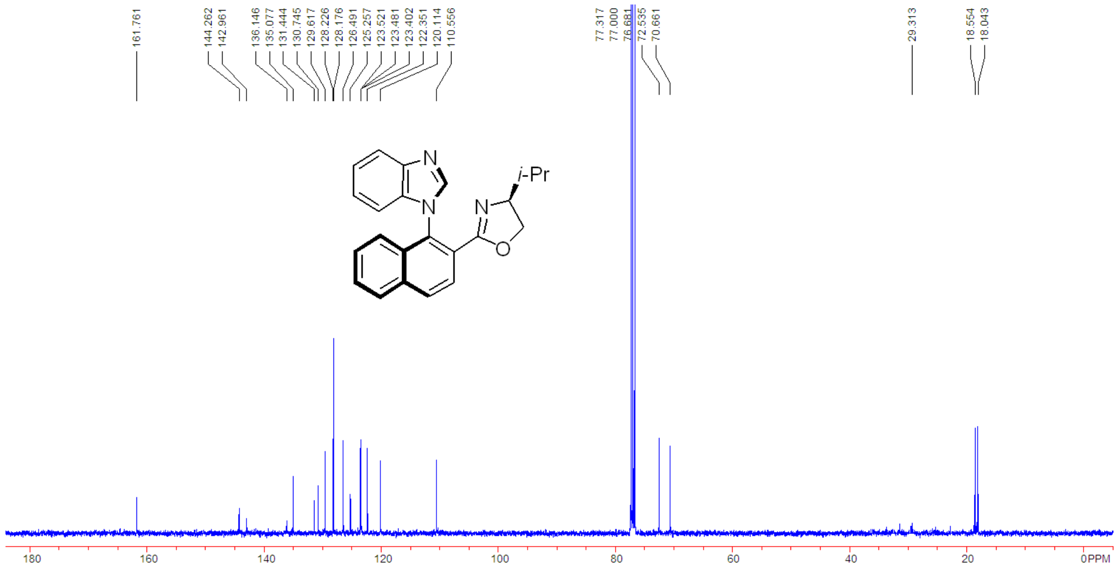

General procedures for the synthesis of axially chiral Au(I) complexes (S_a,S)-16 and (R_a,S)-16.

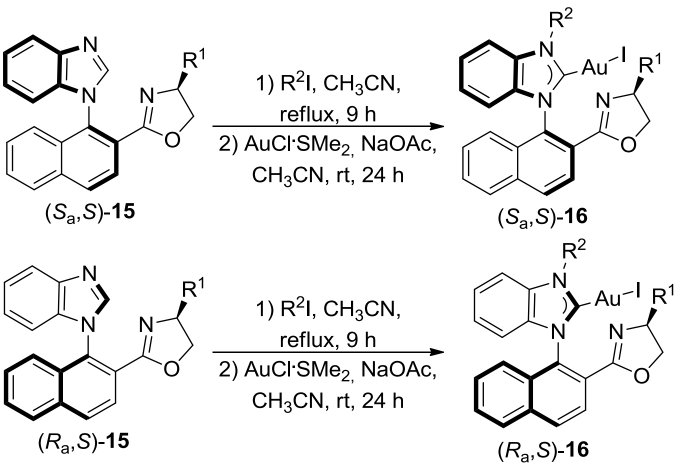

16aa: R^1 = Ph, R^2 = Me

16ab: R^1 = Ph, R^2 = Et

16ba: R^1 = iPr, R^2 = Me

The optically pure compound **15** (1.49 mmol) and R^2I (15 mmol) in CH_3CN (10 mL) were stirred under reflux until the compound **15** was consumed. After cooling to room temperature, volatiles were removed under reduced pressure to obtain the crude benzimidazolium salt **7**. Salt **7**, $AuCl{\cdot}SMe_2$ (0.43 g, 1.47 mmol) and NaOAc (0.25 g, 3.0 mmol) were stirred in CH_3CN (25 mL) for 24 h. The volatiles were then removed under reduced pressure and the residue was purified by silica gel flash column chromatography (dichloromethane/petroleum ether 6/1 to 6/0) to give the corresponding axially chiral Au(I) complex **16**.

Salt **(*S*$_a$,*S*)-7aa**. ^1H NMR ($CDCl_3$, 400 MHz, TMS) δ 4.01 (t, *J* = 8.0 Hz, 1H), 4.32 (s, 3H), 4.70 (t, *J* = 9.6 Hz, 1H), 5.27 (br s, 1H), 6.42 (d, *J* = 8.0 Hz, 2H), 6.99–7.05 (m, 3H), 7.13 (t, *J* = 7.2 Hz, 1H), 7.53–7.74 (m, 6H), 8.08 (d, *J* = 8.4 Hz, 1H), 8.18 (d, *J* = 8.4 Hz, 1H), 8.27 (d, *J* = 8.8 Hz, 1H), 10.58 (s, 1H).

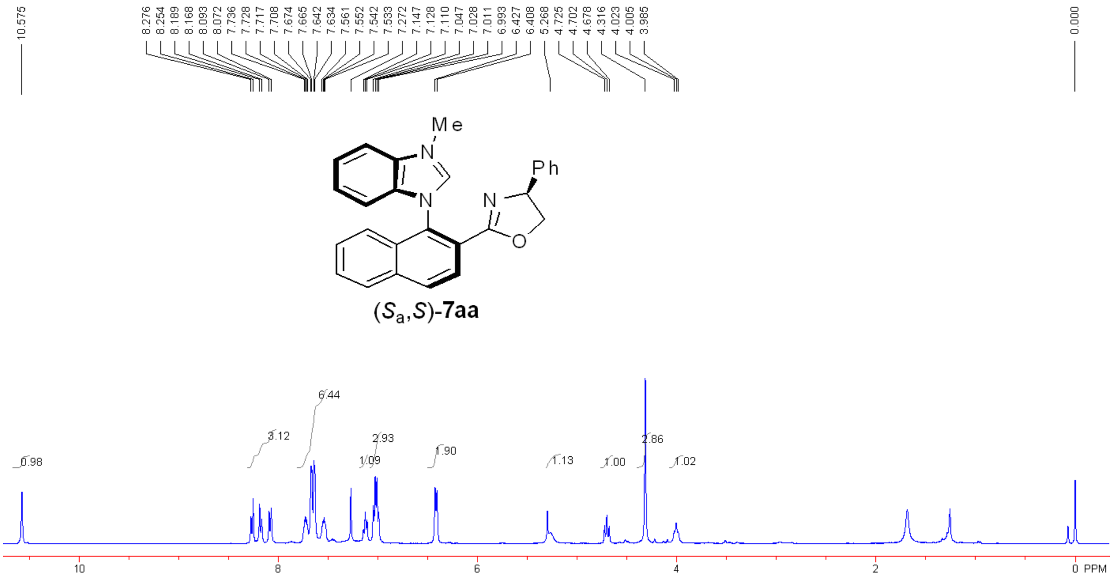

(*S*$_a$,*S*)-16aa. Dark solid, 95% yield; mp >250 °C; $[\alpha]^{20}_D$ = +32 (*c* 0.20, $CHCl_3$); ^1H NMR ($CDCl_3$, 400 MHz, TMS) δ 3.87 (t, *J* = 8.8 Hz, 1H), 4.16 (s, 3H), 4.45 (t, *J* = 8.8 Hz, 1H), 5.15 (t, *J* = 9.2 Hz, 1H), 6.74–6.75 (m, 2H), 6.89 (d, *J* = 7.6 Hz, 1H), 7.16–7.17 (m, 3H), 7.31–7.38 (m, 2H), 7.47–7.54 (m, 3H), 7.65 (t, *J* = 7.6 Hz, 1H), 8.03 (d, *J* = 8.0 Hz, 1H), 8.15 (d, *J* = 8.4 Hz, 1H), 8.27 (d, *J* = 8.8 Hz, 1H); ^{13}C NMR ($CDCl_3$, 100 MHz, TMS) δ 35.0, 69.9, 74.2, 111.2, 112.0, 123.3, 124.0, 124.6, 124.9, 126.2, 126.5, 127.2, 128.4, 128.46, 128.52, 130.46, 130.52, 132.2, 133.3, 135.4, 135.5, 141.7, 161.6, 190.6, 207.0; MS (ESI) *m/z* (%): 728.0 [M + H] (100); HRMS (ESI) calcd. for $C_{27}H_{22}AuIN_3O$ 728.0473; found 728.0474.

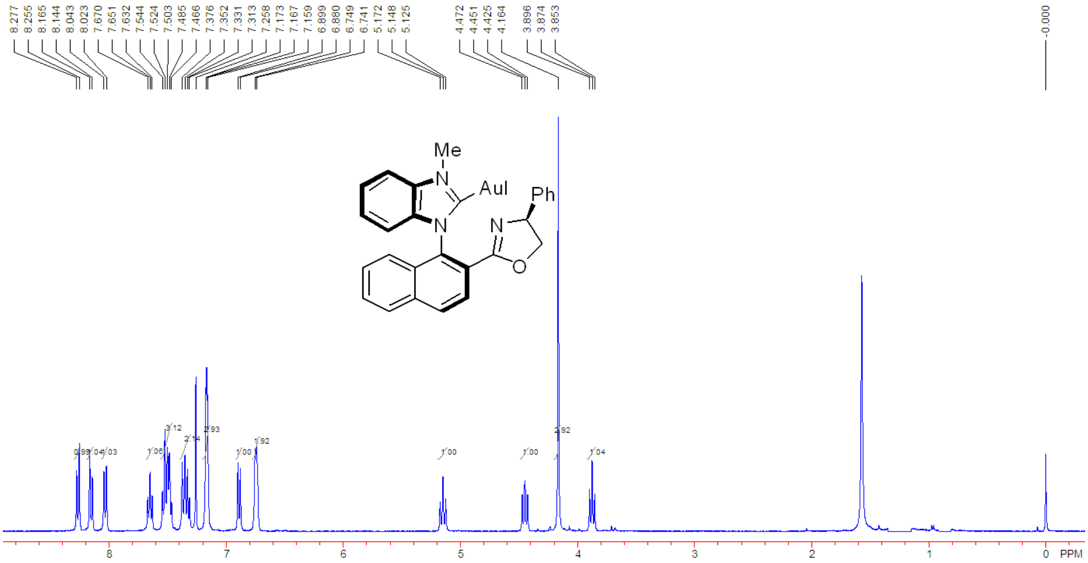

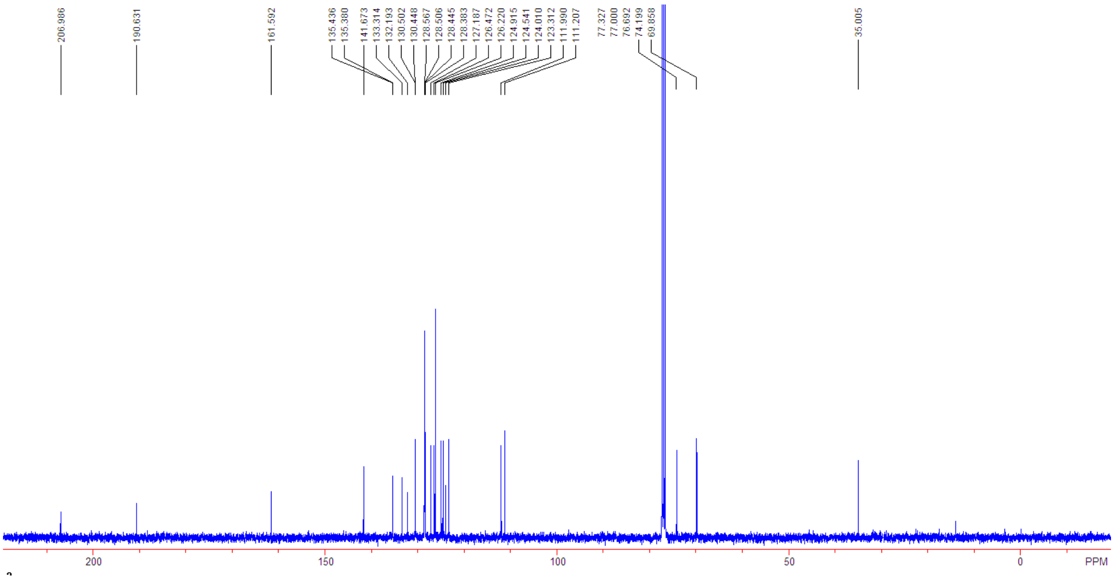

(R_a,*S*)-16aa. Dark solid, 50% yield; mp >250 °C; $[\alpha]^{20}_{D}$ = −36 (*c* 0.30, $CHCl_3$); ^{1}H NMR ($CDCl_3$, 400 MHz, TMS) δ 3.91 (s, 3H), 4.03 (t, *J* = 8.8 Hz, 1H), 4.55 (d, *J* = 8.8, 10.4 Hz, 1H), 5.01 (dd, *J* = 8.8, 10.4 Hz, 1H), 6.87 (d, *J* = 8.0 Hz, 1H), 7.13–7.15 (m, 2H), 7.28–7.36 (m, 5H), 7.45–7.51 (m, 3H), 7.63–7.67 (m, 1H), 8.04 (d, *J* = 8.4 Hz, 1H), 8.16 (d, *J* = 8.8 Hz, 1H), 8.21 (d, *J* = 8.8 Hz, 1H); ^{13}C NMR ($CDCl_3$, 100 MHz, TMS) δ 34.6, 70.4, 73.9, 111.0, 112.0, 123.3, 124.5, 124.9, 126.3, 127.0, 127.5, 128.4, 128.5, 128.7, 130.2, 130.5, 132.3, 133.1, 135.3, 135.5, 141.5, 161.5, 190.2; MS (ESI) *m/z* (%): 728.0 [M + H] (100); HRMS (ESI) calcd. for $C_{27}H_{22}AuIN_3O$ 728.0473; found 728.0474.

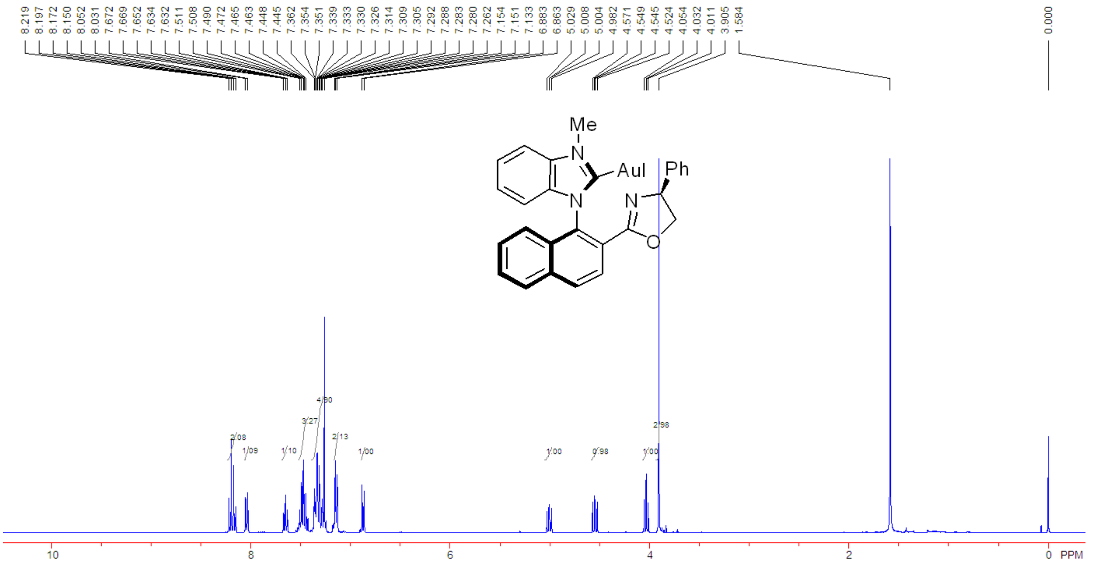

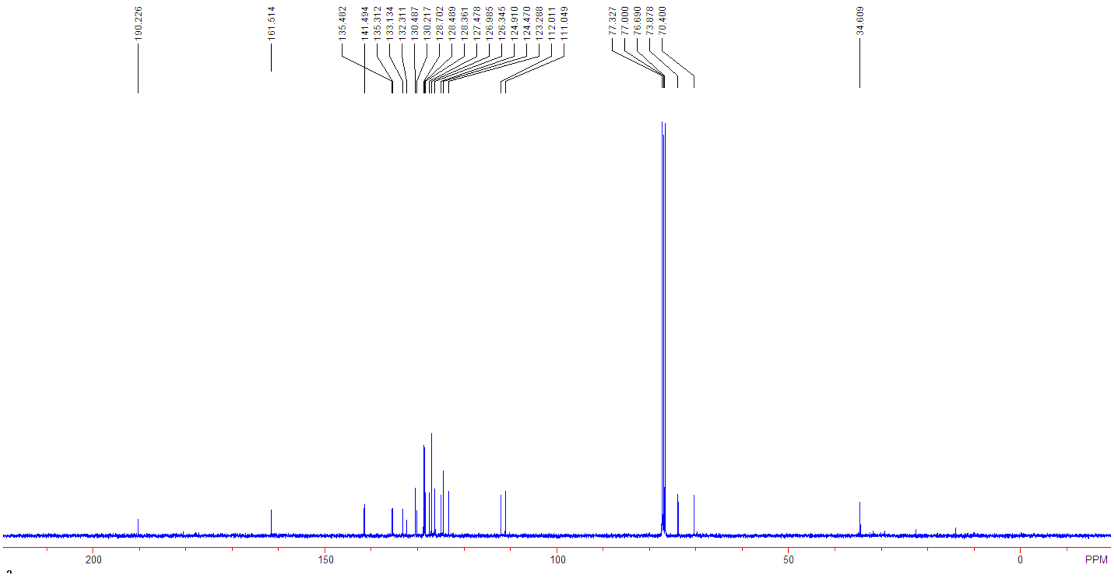

(S_a,S)-16ab. White solid, 89% yield; mp >250 °C; $[\alpha]^{20}_{D}$ +27 (*c* 0.30, $CHCl_3$); ^{1}H NMR ($CDCl_3$, 400 MHz, TMS) δ 1.48 (t, *J* = 7.2 Hz, 3H), 3.85 (t, *J* = 8.8 Hz, 1H), 4.45–4.57 (m, 2H), 4.72–4.78 (m, 1H), 5.13 (t, *J* = 9.6 Hz, 1H), 6.70–6.73 (m, 2H), 6.88 (d, *J* = 8.0 Hz, 1H), 7.15–7.17 (m, 3H), 7.29–7.34 (m, 1H), 7.40 (d, *J* = 8.4 Hz, 1H), 7.45–7.53 (m, 3H), 7.64–7.68 (m, 1H), 8.04 (d, *J* = 8.0 Hz, 1H), 8.16 (d, *J* = 8.4 Hz, 1H), 8.25 (d, *J* = 8.4 Hz, 1H); ^{13}C NMR ($CDCl_3$, 100 MHz, TMS) δ 15.5, 43.6, 69.9, 74.3, 111.3, 112.1, 123.3, 124.0, 124.4, 124.8, 126.3, 126.4, 127.2, 128.40, 128.43, 128.5, 128.6, 130.4, 130.45, 132.2, 132.4, 135.4, 135.6, 141.6, 161.5, 190.1; MS (ESI) *m/z* (%): 742.1 [M + H] (100); HRMS (ESI) calcd. for $C_{28}H_{24}AuIN_3O$ 742.0630; found 742.0634.

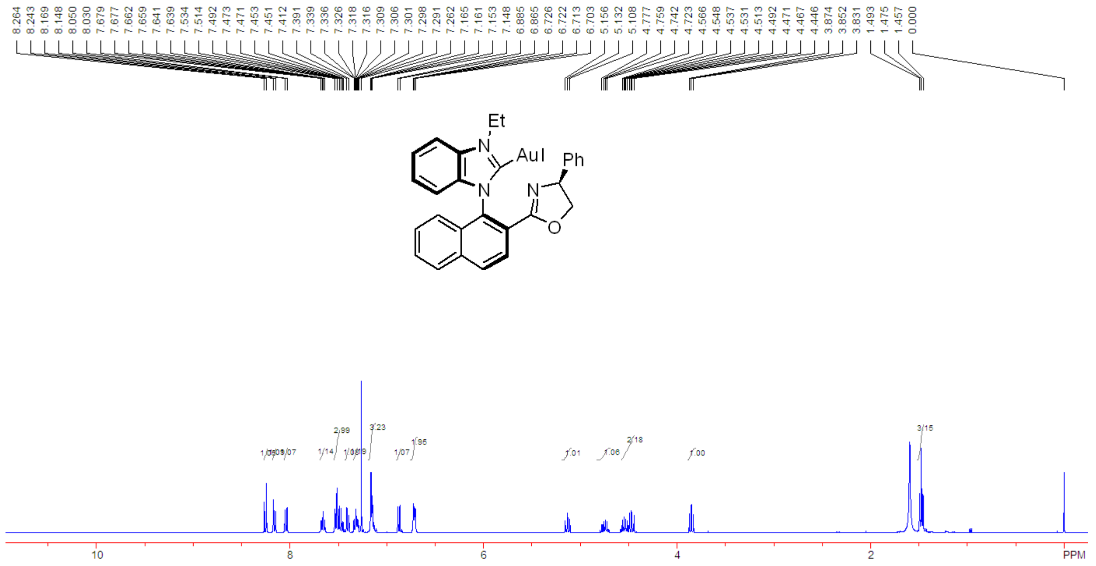

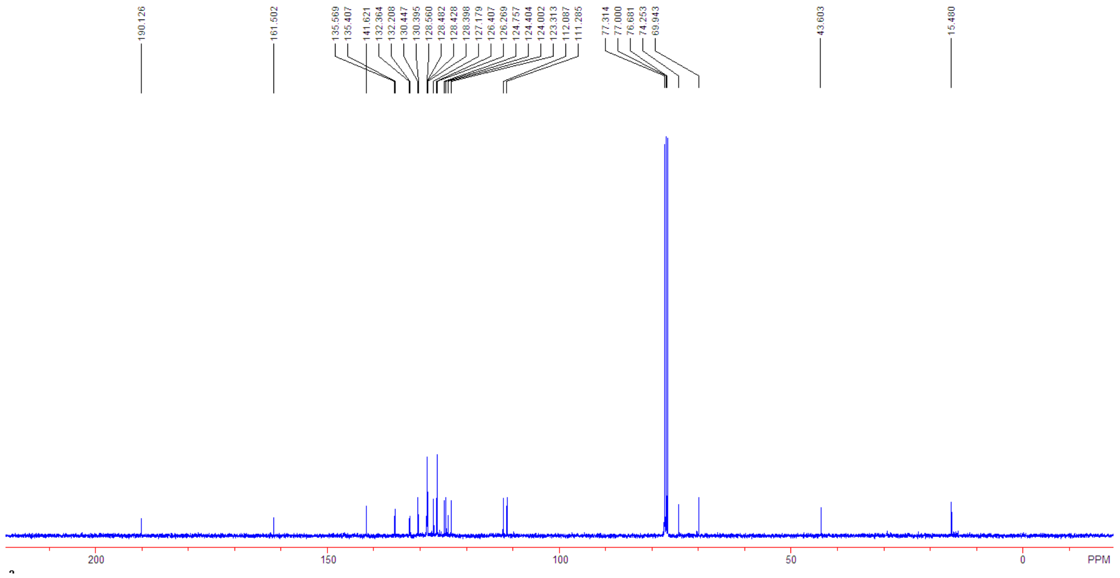

($\boldsymbol{R_a}$,*S*)-16ab. White solid, 48% yield; mp >250 °C; $[\alpha]^{20}_{D}$ −19 (*c* 0.25, $CHCl_3$); ^1H NMR ($CDCl_3$, 400 MHz, TMS) δ 1.33 (t, *J* = 7.6 Hz, 3H), 3.95 (t, *J* = 8.0 Hz, 1H), 4.42-4.57 (m, 3H), 5.01 (dd, *J* = 8.0, 10.0 Hz, 1H), 6.86 (d, *J* = 8.4 Hz, 1H), 7.12 (d, *J* = 7.2 Hz, 2H), 7.24–7.33 (m, 4H), 7.42–7.53 (m, 4H), 7.63–7.67 (m, 1H), 8.04 (d, *J* = 8.0 Hz, 1H), 8.16 (dd, *J* = 2.4, 8.4 Hz, 1H), 8.25 (d, *J* = 8.8 Hz, 1H); ^{13}C NMR ($CDCl_3$, 100 MHz, TMS) δ 15.0, 43.6, 70.5, 74.1, 111.2, 112.1, 123.3, 124.4, 124.7, 126.3, 126.5, 127.0, 127.5, 128.37, 128.44, 128.5, 128.7, 130.3, 130.5, 132.0, 132.4, 135.4, 135.6, 141.5, 161.5, 189.6; MS (ESI) *m/z* (%): 742.1 [M + H] (100); HRMS (ESI) calcd. for $C_{28}H_{24}AuIN_3O$ 742.0630; found 742.0634.

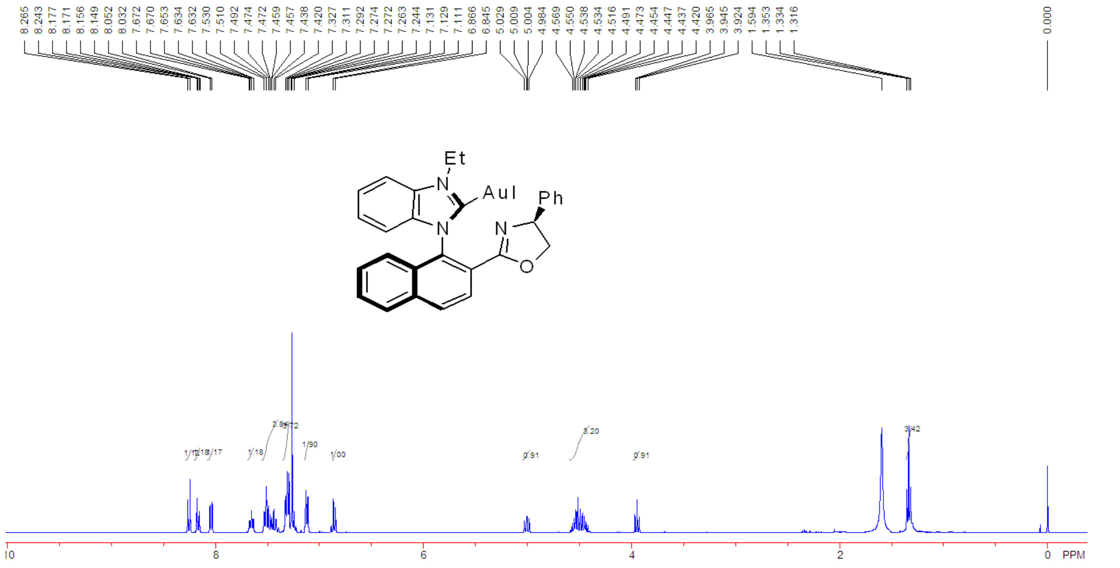

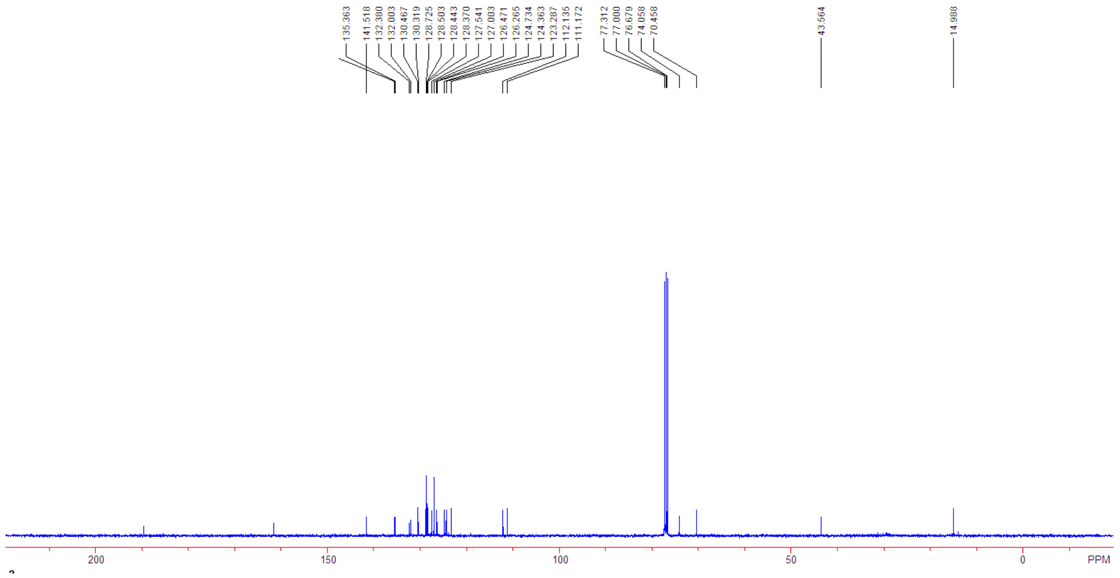

(S_a,S)-16ba. White solid, 90% yield; mp >250 °C; $[\alpha]^{20}_{D}$ = −30 (c 0.25, $CHCl_3$); ^{1}H NMR ($CDCl_3$, 400 MHz, TMS) δ 0.47 (d, J = 6.8 Hz, 3H), 0.58 (d, J = 6.8 Hz, 3H), 0.71 (dd, J = 6.8, 7.6 Hz, 1H), 3.74–3.79 (m, 2H), 4.03–4.09 (m, 1H), 4.23 (s, 3H), 6.82 (d, J = 8.4 Hz, 1H), 7.29 (d, J = 8.0 Hz, 1H), 7.38 (d, J = 8.0 Hz, 1H), 7.44–7.51 (m, 2H), 7.57 (d, J = 8.4 Hz, 1H), 7.63 (t, J = 8.0 Hz, 1H), 8.02 (d, J = 8.0 Hz, 1H), 8.13 (d, J = 8.8 Hz, 1H), 8.17 (d, J = 8.8 Hz, 1H); ^{13}C NMR ($CDCl_3$, 100 MHz, TMS) δ 18.7, 33.1, 34.9, 70.3, 73.3, 110.9, 112.3, 123.2, 124.5, 124.9, 125.0, 126.3, 128.27, 128.30, 128.5, 130.2, 130.4, 131.8, 133.1, 135.2, 135.5, 160.4, 190.3; MS (ESI) m/z (%): 694.1 [M + H] (100); HRMS (ESI) calcd. for $C_{24}H_{24}AuIN_3O$ 694.0630; found 694.0622.

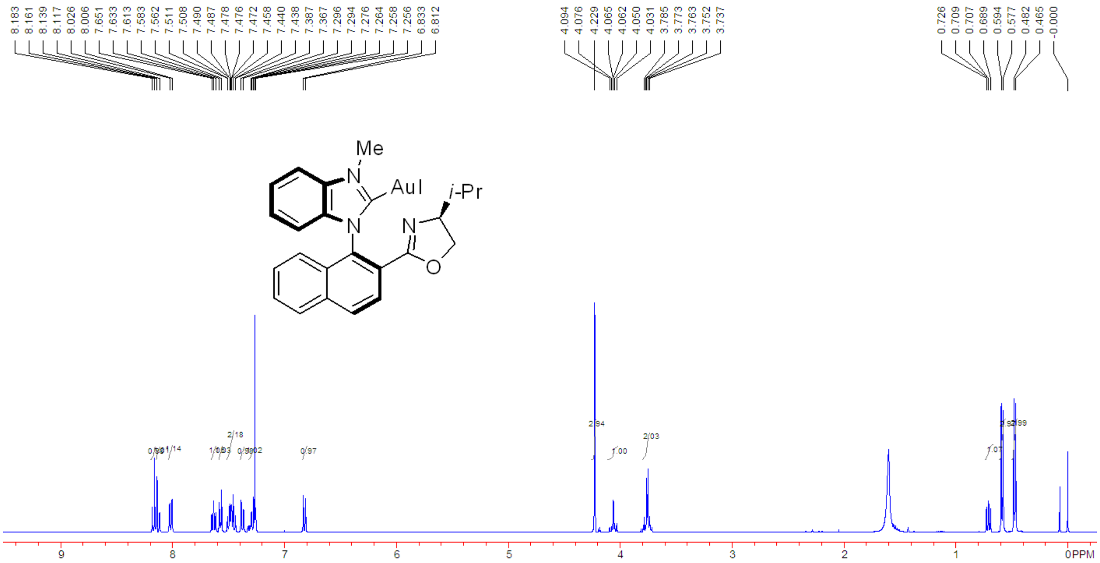

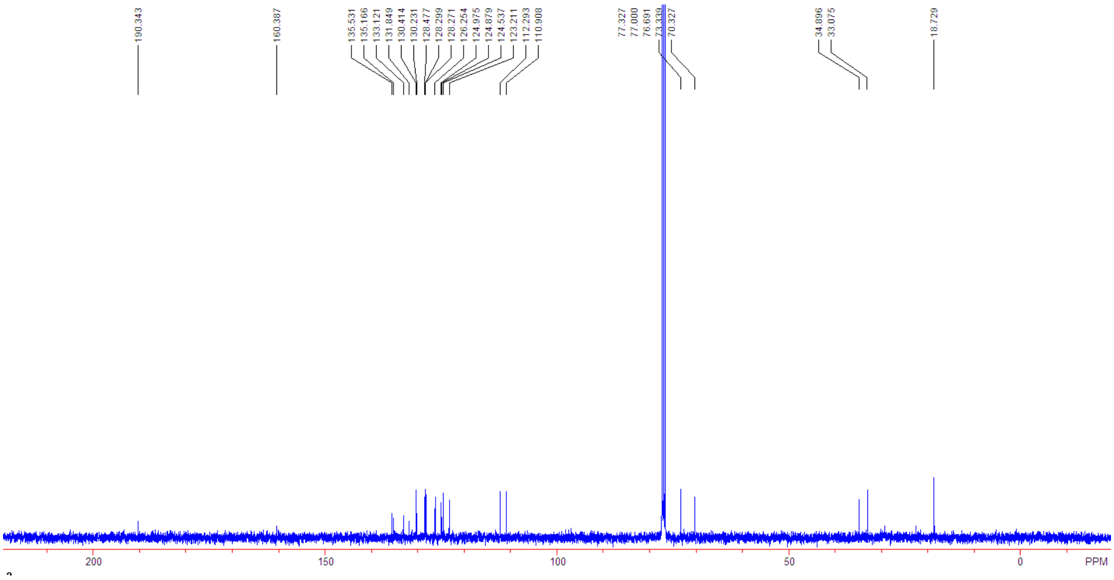

(R_a,S)-16ba. White solid, 56% yield; mp >250 °C; $[\alpha]^{20}_{D}$ −52 (*c* 0.25, $CHCl_3$); ^{1}H NMR ($CDCl_3$, 400 MHz, TMS) δ 0.71 (t, *J* = 6.8 Hz, 6H), 0.88 (t, *J* = 6.8 Hz, 1H), 3.63–3.69 (m, 1H), 3.79 (t, *J* = 8.4 Hz, 1H), 4.18–4.23 (m, 4H), 6.86 (d, *J* = 8.4 Hz, 1H), 7.29–7.33 (m, 2H), 7.45–7.50 (m, 2H), 7.58 (d, *J* = 8.4 Hz, 1H), 7.63 (t, *J* = 8.0 Hz, 1H), 8.02 (d, *J* = 8.0 Hz, 1H), 8.13 (s, 2H); ^{13}C NMR ($CDCl_3$, 100 MHz, TMS) δ 18.0, 18.6, 32.6, 35.0, 69.9, 72.9, 110.9, 111.9, 123.3, 124.2, 124.4, 124.8, 126.3, 128.3, 128.4, 130.4, 130.5, 131.9, 133.3, 135.22, 135.25, 159.8, 190.6; MS (ESI) *m/z* (%): 694.1 [M + H] (100); HRMS (ESI) calcd. for $C_{24}H_{24}AuIN_3O$ 694.0630; found 694.0622.

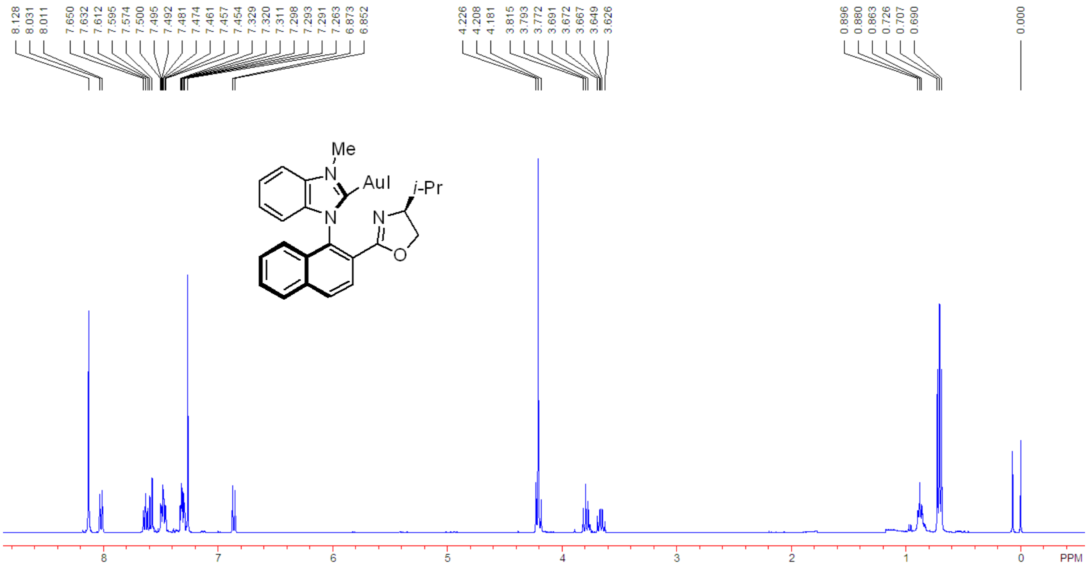

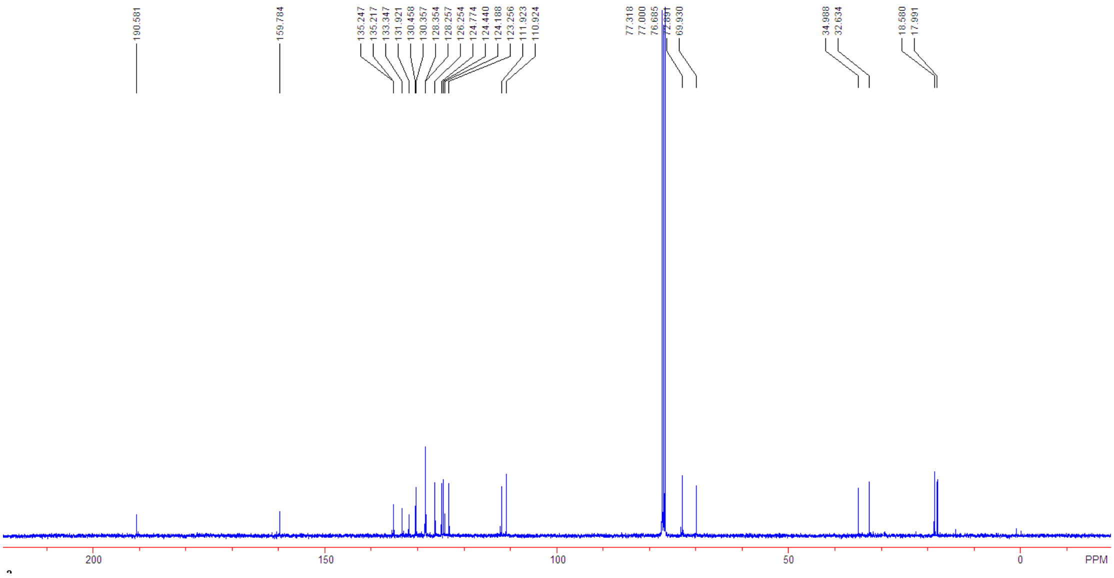

References

1. Wessig, P.; Müller, G. *Aust. J. Chem.* **2008,** *61*, 569–572. doi:10.1071/CH08141
